# N‐homocysteinylation of α‐synuclein promotes its aggregation and neurotoxicity

**DOI:** 10.1111/acel.13745

**Published:** 2022-11-27

**Authors:** Lingyan Zhou, Tao Guo, Lanxia Meng, Xingyu Zhang, Ye Tian, Lijun Dai, Xuan Niu, Yiming Li, Congcong Liu, Guiqin Chen, Chaoyang Liu, Wei Ke, Zhaohui Zhang, Anyu Bao, Zhentao Zhang

**Affiliations:** ^1^ Department of Neurology Renmin Hospital of Wuhan University Wuhan China; ^2^ Department of Pathology and Laboratory Medicine Emory University School of Medicine Atlanta Georgia USA; ^3^ Research Center for Environment and Health Zhongnan University of Economics and Law Wuhan China; ^4^ Department of Clinical Laboratory Renmin Hospital of Wuhan University Wuhan China; ^5^ TaiKang Center for Life and Medical Science Wuhan University Wuhan China

**Keywords:** homocysteine thiolactone, N‐homocysteinylation, Parkinson's disease

## Abstract

The aggregation of α‐synuclein plays a pivotal role in the pathogenesis of Parkinson's disease (PD). Epidemiological evidence indicates that high level of homocysteine (Hcy) is associated with an increased risk of PD. However, the molecular mechanisms remain elusive. Here, we report that homocysteine thiolactone (HTL), a reactive thioester of Hcy, covalently modifies α‐synuclein on the K80 residue. The levels of α‐synuclein K80Hcy in the brain are increased in an age‐dependent manner in the TgA53T mice, correlating with elevated levels of Hcy and HTL in the brain during aging. The N‐homocysteinylation of α‐synuclein stimulates its aggregation and forms fibrils with enhanced seeding activity and neurotoxicity. Intrastriatal injection of homocysteinylated α‐synuclein fibrils induces more severe α‐synuclein pathology and motor deficits when compared with unmodified α‐synuclein fibrils. Increasing the levels of Hcy aggravates α‐synuclein neuropathology in a mouse model of PD. In contrast, blocking the N‐homocysteinylation of α‐synuclein ameliorates α‐synuclein pathology and degeneration of dopaminergic neurons. These findings suggest that the covalent modification of α‐synuclein by HTL promotes its aggregation. Targeting the N‐homocysteinylation of α‐synuclein could be a novel therapeutic strategy against PD.

## Graphical abstract

Hyperhomocysteinemia is associated with an increased risk of PD. However, the molecular mechanisms remain elusive. HTL, a reactive thioester of Hcy, covalently modifies a‐synuclein at the K80 residue. The N‐homocysteinylation of a‐synuclein stimulates its aggregation and neurotoxicity. Our findings suggest that targeting the N‐homocysteinylation of a‐synuclein could be a novel therapeutic strategy against PD.

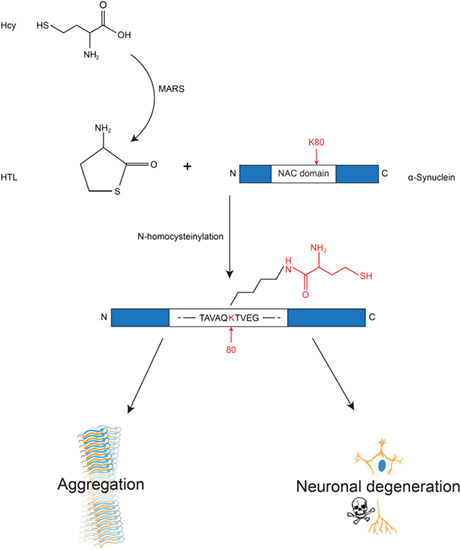

**Abbreviations**
AAVs: adeno‐associated viruses
CBS: Cystathionine β‐synthase
Hcy: Homocysteine
HTL: Homocysteine thiolactone
IHC: immunohistochemistry
K80: lysine 80
KHcy: homocysteinylation
LBs: Lewy bodies
LNs: Lewy neurites
MARS: Methionine‐tRNA synthetase
Met: methionine
PD: Parkinson's disease
PFFs: Pre‐formed fibrils
PK: Proteinase K
pS129: Phosphorylated α‐syn
SNpc: Substantia nigra pars compacta
TgA53T: α‐Syn A53T transgenic
TH: Tyrosine hydroxylase
ThS: Thioflavin S
ThT: Thioflavin T
α‐syn: α‐Synuclein

## 1 INTRODUCTION

Parkinson's disease (PD) is one of the most common neurodegenerative diseases caused by the loss of dopaminergic neurons in the substantia nigra pars compacta (SNpc). Pathologically, PD is characterized by the formation of intraneuronal inclusions called Lewy bodies (LBs) and Lewy neurites (LNs), which are mainly composed of aggregated α‐synuclein (α‐syn) (Fares et al., 2021). Under physiological conditions, α‐syn is a natively unstructured synaptic protein that is mainly expressed in the presynaptic nerve terminals (Bartels et al., 2011). Converging evidence indicates that the toxicity of α‐syn is related to its aggregation (Araki et al., 2019). However, the molecular mechanisms that drive the aggregation of α‐syn during the onset of PD have not been completely identified.

Multiple epidemiological and clinical studies reported that high level of homocysteine (Hcy) is associated with an increased risk of PD (Bakeberg et al., 2019; Licking et al., 2017). Interestingly, the levels of Hcy gradually increase with age (Mattson et al., 2002). High level of Hcy has been implicated in several human diseases including stroke (Carlsson, 2007), coronary artery disease (Foody et al., 2000), and Alzheimer's disease (Zhuo & Pratico, 2010). Several potential mechanisms have been proposed to explain the biological links between Hcy elevation and the onset of diseases. Hcy has been reported to induce inflammation (Elsherbiny et al., 2020), microvascular damage (Muzurovic et al., 2021), and autoimmune responses (Lazzerini et al., 2007). However, the causative role of Hcy in PD pathogenesis remains to be elucidated.

Hcy is a byproduct of the methionine metabolism pathway. Methionine supplements boost Hcy levels (Zhang et al., 2019). Homocysteine thiolactone (HTL) is a reactive intermediate of Hcy generated by methionine‐tRNA synthetase (MARS). Recently, several studies reported that HTL covalently modifies certain lysine residues in proteins, a process known as N‐homocysteinylation. The N‐homocysteinylation of proteins results in the alteration of their structure and function (Jakubowski, 2011; Sikora et al., 2010). For example, N‐homocysteinylation of bovine serum albumin (BSA) induces it to form amyloid‐like structures (Paoli et al., 2010). α‐Synuclein is subjected to extensive post‐transcriptional modifications, including O‐GlcNAcylation (Levine et al., 2019), oxidation (Ponzini et al., 2019), and glycation (Vicente Miranda et al., 2017). These modifications regulate its oligomerization, polymerization, and toxicity in vivo. Since the levels of Hcy are increased in PD patients (Saadat et al., 2018), we speculated that HTL might modify α‐syn to bring about the onset of PD.

In the current work, we identified that the K80 residue of α‐syn undergoes N‐homocysteinylation, which enhances α‐syn aggregation and forms aggregates with enhanced seeding activity and neurotoxicity in vitro and in vivo. Blocking the N‐homocysteinylation of α‐syn at K80 attenuates α‐syn pathology. Therefore, we demonstrate that N‐homocysteinylation mediates α‐syn pathology, promoting the onset and progression of PD.

## 2 RESULTS

### 2.1 α‐Syn is covalently modified by HTL

**FIGURE 1:**
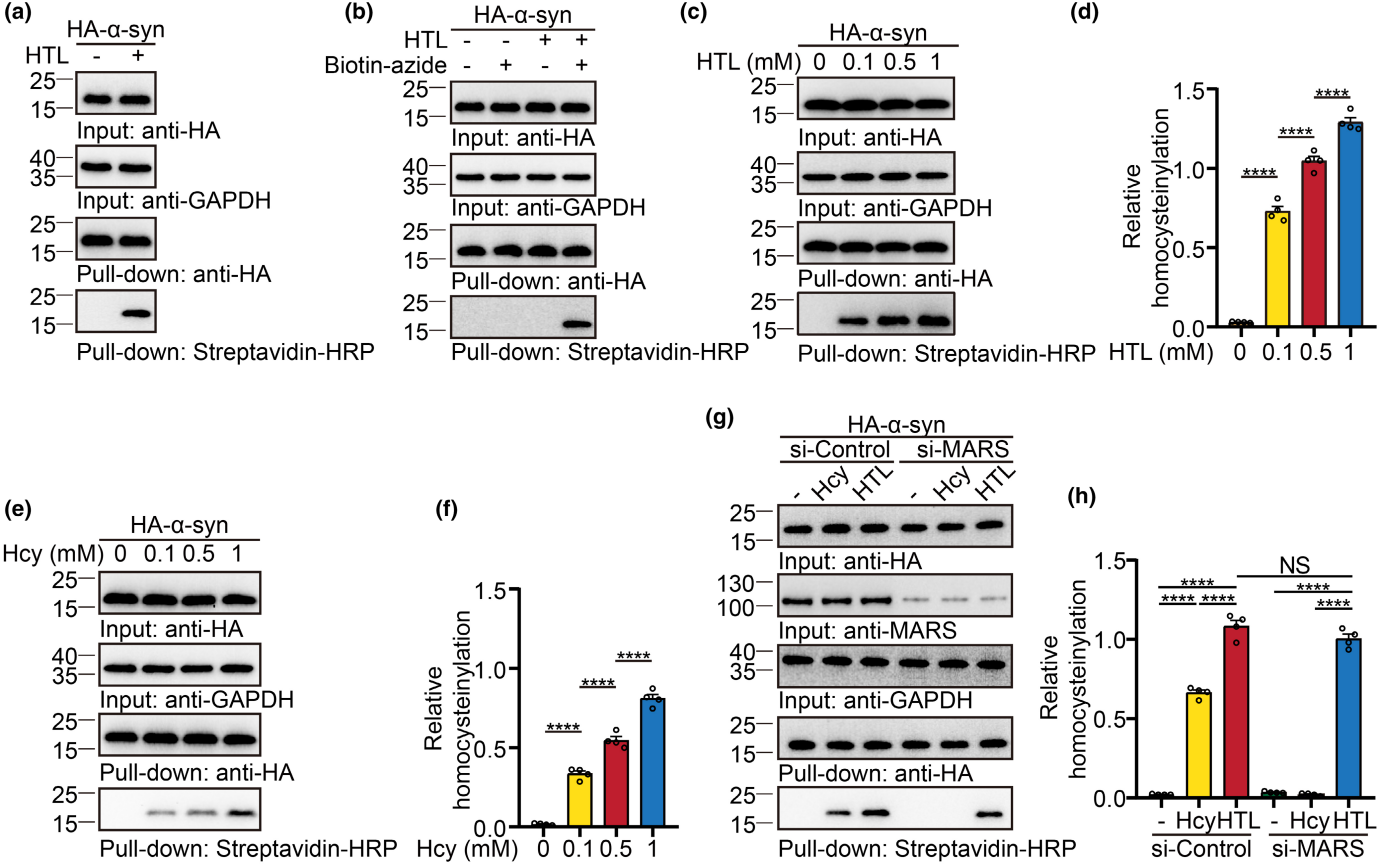
α‐Syn is N‐homocysteinylated. HEK293 cells were transfected with HA‐α‐syn, followed by treatment with vehicle, Hcy, or HTL. HA‐α‐syn were purified using HA beads from the cell lysates and labeled by the chemoselective reactions. (a) Chemoselective labeling of HA‐α‐syn‐transfected HEK293 cells incubated with vehicle or HTL (0.1 mM) for 12 h. (b) Verification of the selectivity of the biotin‐azide probe. (c, d) HTL induces α‐syn homocysteinylation in a concentration‐dependent manner. (e, f) Hcy induces α‐syn homocysteinylation in a concentration‐dependent manner. (g, h) Knockdown of MARS abolishes α‐syn homocysteinylation induced by Hcy. Data are shown as mean ± SEM. *n* = 4 independent experiments. ****p < 0.0001, NS, not significant

Protein N‐homocysteinylation can be specifically labeled with azide probes (Chen et al., 2019). To explore whether α‐syn undergoes N‐homocysteinylation, we treated HEK293 cells expressing HA‐tagged α‐syn with HTL and performed chemoselective reaction using a biotin‐azide probe. Immunoblotting revealed that α‐syn was homocysteinylated in the presence of HTL (Figure 1a). No signal was detected in the absence of HTL or biotin‐azide probe, confirming the specificity and selectivity of the probe (Figure 1b). Furthermore, HTL induced the homocysteinylation of α‐syn in a concentration‐dependent manner (Figure 1c,d). HTL is the reactive thioester of Hcy produced by the enzyme MARS in an error editing reaction. We further tested whether Hcy also causes the homocysteinylation of α‐syn in cells. Treatment with Hcy induced α‐syn homocysteinylation (KHcy) in a concentration‐dependent manner (Figure 1e,f). Interestingly, knockdown of MARS abolished α‐syn homocysteinylation induced by Hcy, but not that induced by HTL, indicating the conversion of Hcy to HTL is required for α‐syn homocysteinylation (Figure 1g,h). Together, our findings suggest that the Hcy metabolite HTL mediates α‐syn homocysteinylation.

### 2.2 Lysine 80 is the major residue of α‐syn homocysteinylation

**FIGURE 2:**
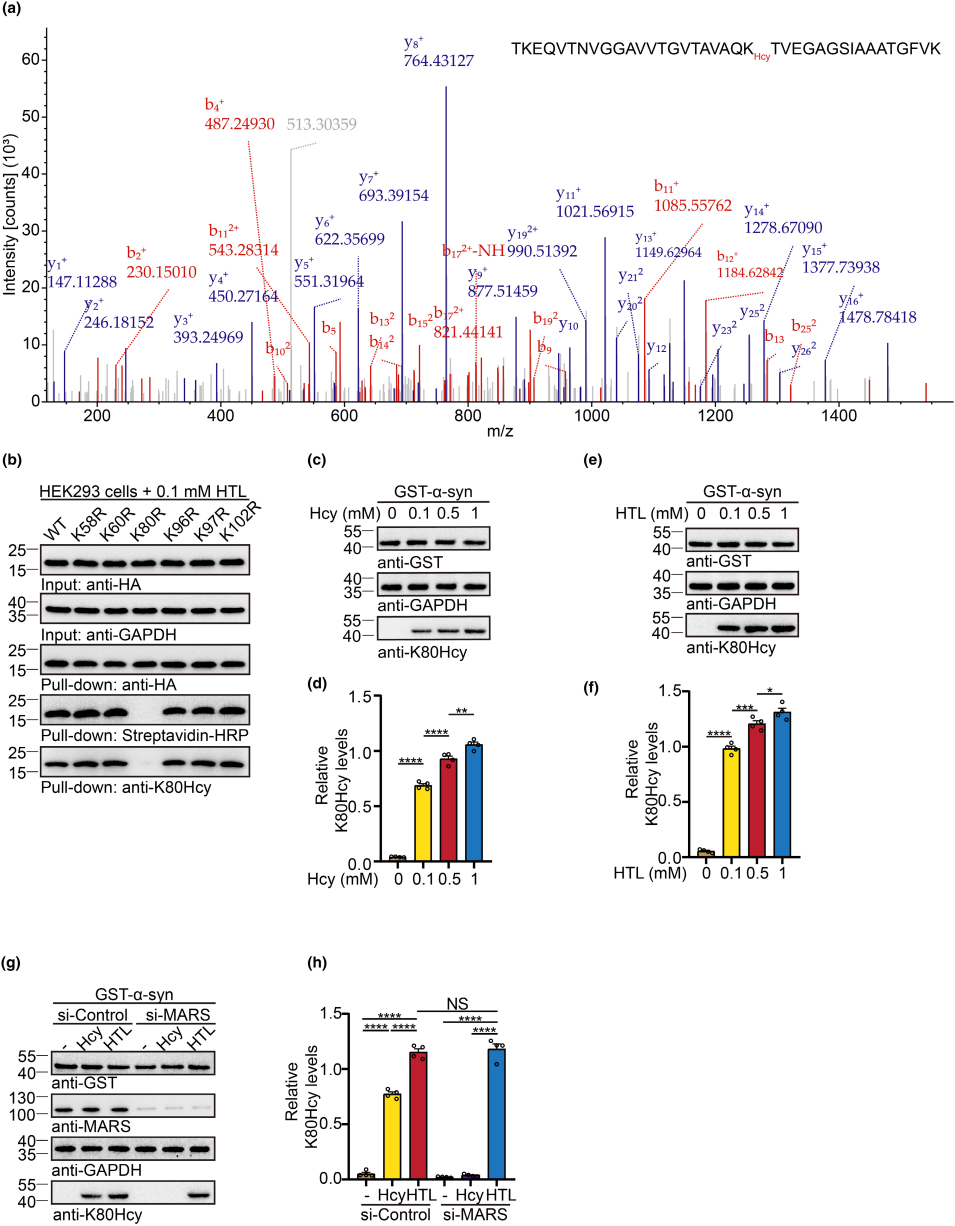
K80 is the major homocysteinylation site on α‐syn. (a) A representative spectrum of LC–MS/MS fragmentation containing K80 homocystylation. (b) K80R mutation abolishes the homocysteinylation of α‐syn. (c, d) Levels of α‐syn K80Hcy in HEK293 cells treated with different concentrations of Hcy. (e, f) Levels of α‐syn K80Hcy in HEK293 cells treated with different concentrations of HTL. (g, h) Knockdown of MARS abolishes Hcy‐induced α‐syn K80Hcy in HEK293 cells. Data are shown as mean ± SEM. *n* = 4 independent experiments. *p < 0.05, **p < 0.01, ***p < 0.001, ****p < 0.0001, NS, not significant

To identify the N‐homocysteinylation site on α‐syn, we transfected GST‐tagged α‐syn into HEK293 cells, treated with HTL, and purified GST‐α‐syn. MS identified multiple homocysteinylated lysine residues throughout the α‐syn sequence (Table S1), with lysine 80 (K80) as the major modified residue (Figure 2a). To confirm the modification of lysine residues, we generated point mutations that replace lysine with arginine (K58R, K60R, K80R, K96R, K97R, K102R). K80R mutation substantially abolished α‐syn homocysteinylation, implying that K80 is the major site of α‐syn homocysteinylation (Figure 2b). We generated a polyclonal antibody (anti‐K80Hcy) by immunizing the rabbits with synthetic α‐syn peptide (amino acids 75–84) containing homocysteinylated lysine at K80. Dot blots showed that anti‐K80Hcy antibody preferentially recognized α‐syn K80Hcy peptide (Figure S1). The K80R mutation completely abolished the K80Hcy signals, further confirming the specificity of the anti‐K80Hcy antibody (Figure 2b). In HEK293 cells transfected with GST‐α‐syn, both Hcy and HTL induced homocysteinylation of the K80 residue as detected with the anti‐K80Hcy antibody (Figure 2c–f). In agreement with the results using azide probes (Figure 1g,h), knockdown of the enzyme MARS abrogated the modification of K80 by Hcy, but not that by HTL (Figure 2g,h). These results indicate that K80 is the major homocysteinylation site on α‐syn.

### 2.3 α‐Syn K80Hcy is increased in the brain in an age‐dependent manner

**FIGURE 3:**
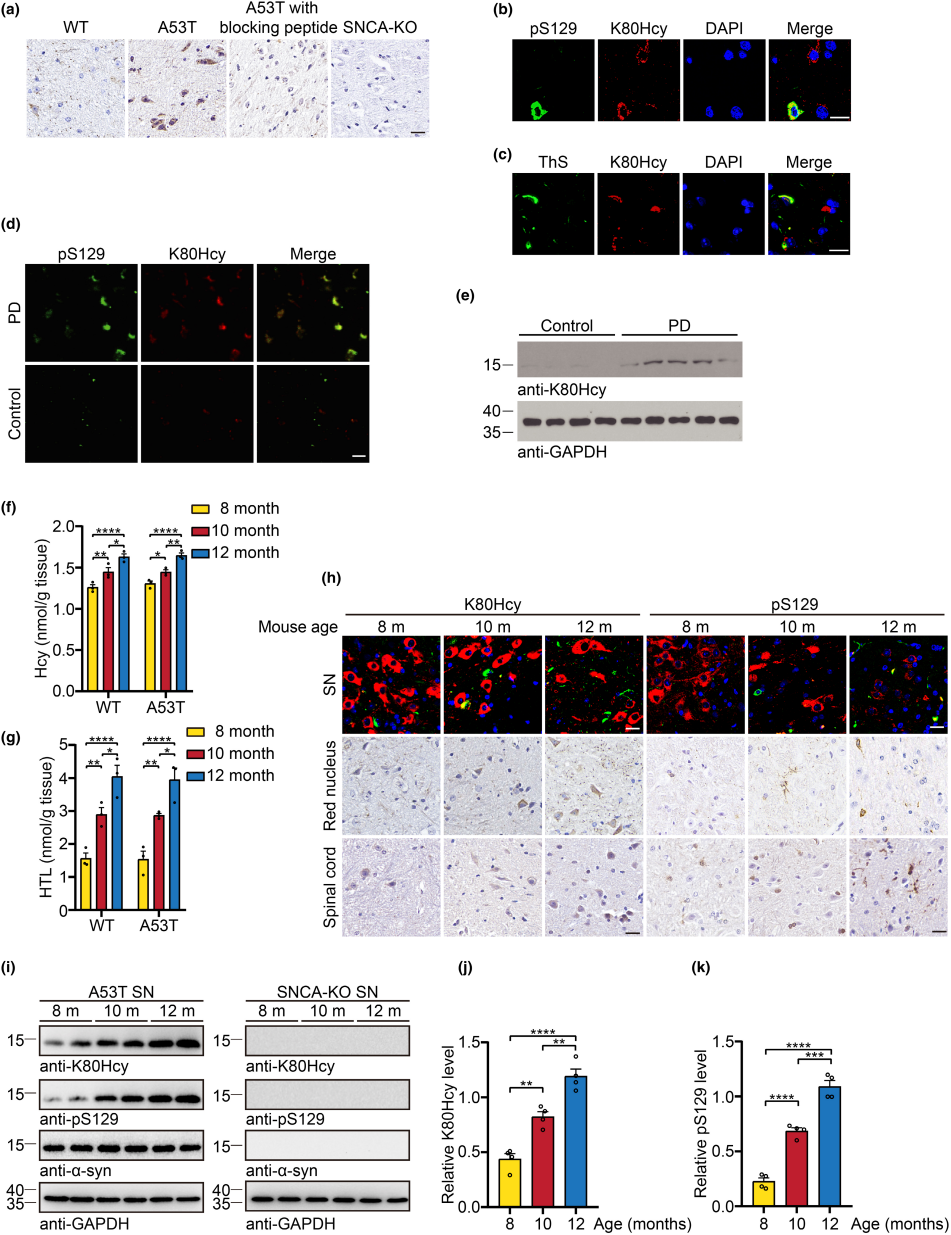
α‐Syn K80Hcy is increased in TgA53T mice in an age‐dependent manner. (a) Immunohistochemistry of the α‐syn K80Hcy in SN sections from 12‐month‐old WT, TgA53T, and *Snca* KO mice. (b) Double immunofluorescence of K80Hcy and pS129 on SN sections from 12‐month‐old TgA53T mice. (c) Colocalization of K80Hcy with Thioflavin S (ThS) on SN sections from 12‐month‐old TgA53T mice. (d) Immunofluorescent staining of K80Hcy and pS129 on the SN sections from PD patients. (e) Western blot of K80Hcy in the SN tissues from PD and control subjects. (f, g) LC/MS analysis of Hcy (f) or GC/MS analysis of HTL (g) in the striatum of TgA53T mice and their wild‐type littermates at different ages. (h) Immunostaining of α‐syn K80Hcy or pS129 in the SN, red nucleus, and spinal cord from different‐age TgA53T mice. (i–k) Western blot quantification of α‐syn K80Hcy and pS129 in the SN of TgA53T and *Snca* KO mice at different ages. Data are shown as mean ± SEM. *n* = 3 (f, g), 4 (i–k) independent experiments. *p < 0.05, **p < 0.01, ***p < 0.001, ****p < 0.0001. Scale bar is 20 μm.

To test whether α‐syn K80Hcy is present in the brain, we performed immunohistochemistry (IHC) using anti‐K80Hcy antibody. α‐Syn K80Hcy was detected in the α‐syn A53T transgenic (TgA53T) mice. The signals were blocked by pre‐incubation with the K80Hcy peptide. No signal was detected in brain sections from *Snca* KO mice, further confirming the specificity of the anti‐K80Hcy antibody (Figure 3a). Moreover, immunofluorescence found that K80Hcy colocalized with phosphorylated α‐syn (pS129), the marker of α‐syn inclusions (Figure 3b). Furthermore, the K80Hcy signals were also positive for thioflavin S (ThS) staining, suggesting the aggregated α‐syn is homocysteinylated (Figure 3c). Remarkably, immunofluorescent staining of the SN sections from PD patients also confirmed that K80Hcy colocalized with pS129 (Figure 3d). Immunoblotting with α‐syn K80Hcy antibody revealed that α‐syn K80Hcy was elevated in the SN from PD brains (Figure 3e). ELISA showed that K80 modification in standard was recognized by anti‐K80Hcy antibody in a concentration‐dependent manner, while the anti‐K80Hcy antibody did not react with the α‐syn K80R mutant or unmodified α‐syn (Figure S2a). ELISA showed that the percentage of α‐syn K80Hcy in control subjects was 1.63%, while that in PD patients was about 5.57% (Figure S2b). Aging is the most important risk factor for PD. We found that the concentrations of both Hcy and HTL in the mouse brain samples increased in an age‐dependent manner (Figure 3f,g). Consistently, both α‐syn K80Hcy and pS129 immunoreactivity were escalated in the TgA53T mice in an age‐dependent manner (Figure 3h). These observations were recapitulated by Western blot analysis (Figure 3i–k). ELISA showed that 1.99%, 3.47%, and 6.05% of α‐syn was modified in TgA53T mice at 8, 10, and 12 months of age, respectively (Figure S2c). Thus, the age‐dependent increase of Hcy and HTL in the brain is accompanied by the accumulation of homocysteinylated α‐syn.

### 2.4 Homocysteinylation of α‐syn facilitates its fibrillization

**FIGURE 4:**
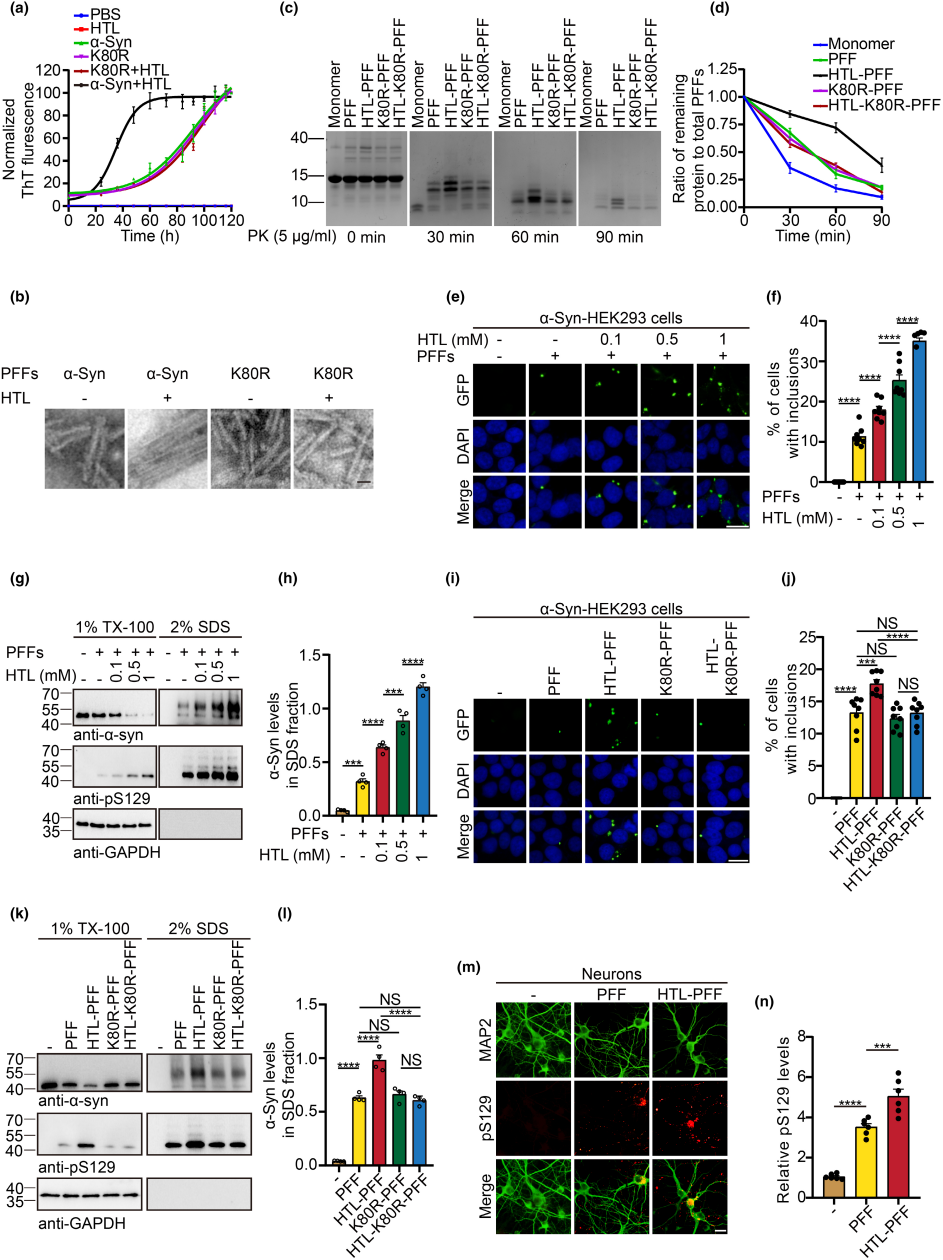
HTL potentiates α‐syn aggregation. (a) Kinetics of α‐syn aggregation in the presence or absence of HTL. Data were normalized to the highest signal. (b) Electron microscopy images of α‐syn fibrils formed by WT and K80R mutant α‐syn in the presence or absence of HTL. Scale bar is 200 nm. (c, d) Proteinase K digestion of WT and K80R mutant α‐syn fibrils formed in the presence or absence of HTL. Quantification represents the ratio of remaining protein to total PFFs. (e–h) α‐Syn‐HEK293 cells were exposed to HTL for 12 h, then transduced with α‐syn PFFs (140 ng/ml final concentration) and incubated for another 24 h. (e, f) Images and quantification of insoluble α‐syn inclusions. (g, h) Western blot analysis of Syn211 and pS129 in Triton X‐100‐soluble and SDS‐soluble fractions. (i–l) WT or K80R mutant α‐syn PFFs were induced to aggregate into PFFs in the presence or absence of HTL. (i) The seeding activity of different PFFs was tested in α‐syn‐HEK293 cells. (j) Quantification of the percentage of the cells with insoluble α‐syn inclusions. (k, l) Western blot analysis of Syn211 and pS129 in Triton X‐100‐soluble and SDS‐soluble fractions. (m, n) Primary neurons were treated with α‐syn PFFs or HTL‐α‐syn PFFs. Immunofluorescence shows α‐syn phosphorylation. *n* = 3 (a), 4 (c, d), 8 (e, f), 4 (g, h), 8 (i, j), 4 (k, l), 6 (m, n) independent experiments. All data are shown as mean ± SEM. ***p < 0.001, ****p < 0.0001. Scale bar is 20 μm.

To explore the effect of homocysteinylation on α‐syn fibrillization, we monitored the kinetics of α‐syn fibrillation via Thioflavin T (ThT) assay. HTL dramatically facilitated α‐syn fibrillization with decreased lag time. However, the fibrillization of K80R mutant α‐syn was not affected by HTL (Figure 4a, Table S2). Electron microscopy revealed that the fibrils formed in the presence of HTL were more condensed than the control fibrils (Figure 4b). To determine the properties of α‐syn pre‐formed fibrils (PFFs), we conducted limited proteolysis of fibrils with proteinase K (PK) and pronase. HTL‐modified α‐syn PFFs were more resistant to digestion with protease K or pronase than control α‐syn PFFs (Figure 4c,d, Figure S3a).

To further investigate the impact of K80 homocysteinylation on α‐syn fibrillization in cells, we used HEK293 cells stably transfected with α‐synuclein‐GFP (α‐syn‐HEK293 cells) as reporter cells. Transduction of the reporter cells with α‐syn PFFs induces the formation of insoluble α‐syn inclusions (Yan et al., 2022). ELISA analysis using the anti‐K80Hcy antibody showed that 4.94% of α‐syn was modified in α‐syn‐HEK293 cells exposure to 0.1 mM HTL (Figure S3b). Pre‐treatment of the reporter cells with HTL dose‐dependently potentiated the formation of α‐syn aggregates induced by α‐syn PFFs (Figure 4e,f). The aggregates colocalized with ubiquitin, a marker of Lewy bodies (Figure S3c). Fractionation analysis revealed that HTL treatment decreased the levels of α‐syn in the Triton X‐100‐soluble fraction, while increased that in the insoluble fraction (Figure 4g,h). Moreover, HTL induced α‐syn phosphorylation in the absence of α‐syn PFFs in α‐syn‐HEK293 cells and neurons (Figure S3d‐g). Similar to HTL, Hcy also resulted in an elevation of α‐syn aggregation in α‐syn‐HEK293 cells (Figure S4a‐d). Knocking down of MARS in α‐syn‐HEK293 cells prevented the elevation of α‐syn aggregation induced by Hcy treatment, but not by HTL (Figure S4e‐h), indicating that the conversion of Hcy to HTL is required for it to promote α‐syn aggregation in cells.

To further confirm the effect of α‐syn K80 modification on its aggregation, we treated HEK293 cells expressing wild‐type or K80R mutant α‐syn with Hcy or HTL, and then transduced the cells with α‐syn PFFs. Hcy and HTL promoted the aggregation of wild‐type α‐syn, but not the K80R mutant α‐syn (Figure S5a,b). The results were further validated by Western blot analysis of the soluble and insoluble α‐syn species (Figure S5c‐f).

### 2.5 Homocysteinylated α‐syn PFFs are more prone to seed soluble α‐syn in vitro

To investigate the effect of K80Hcy on the seeding activity of α‐syn fibrils, we generated PFFs from wild‐type and K80R mutant α‐syn in the presence or absence of HTL, and then transduced α‐syn‐HEK293 cells with these PFFs. ELISA assay showed that 10.63% of α‐syn was modified by K80Hcy in α‐syn PFFs generated from wild‐type α‐syn in the presence of HTL (Figure S6a). Coomassie Blue staining confirmed that equal amounts of fibrils were used (Figure S6b). While the PFFs generated from K80R mutant α‐syn displayed comparable seeding capability to α‐syn PFFs. They were resistant to HTL's increasing effect in seeding α‐syn aggregation (Figure 4i,j). Fractionation analysis found that HTL‐modified α‐syn PFFs show enhanced seeding activity, which was abolished by K80R mutation (Figure 4k,l). When transduced to primary neurons, the homocysteinylated α‐syn PFFs were more potent to induce α‐syn phosphorylation (Figure 4m,n). These results indicate that K80Hcy potentiates α‐syn aggregation, and results in α‐syn fibrils with enhanced seeding activity.

### 2.6 Homocysteinylated α‐syn fibrils show enhanced seeding activity and neurotoxicity in vivo

To investigate the effect of homocysteinylation on the neurotoxicity of α‐syn fibrils, we injected α‐syn PFFs and HTL‐modified PFFs into the striatum of wild‐type mice brains and monitored α‐syn pathology 6 months after injection. Compared with α‐syn PFFs, HTL‐PFFs induced more deposition of pS129 (Figure S7a). HTL‐α‐syn PFFs caused an increased accumulation of high molecular weight α‐syn species (Figure S7b‐g). Furthermore, the expression of the microglial marker IBA1 and astrocyte marker GFAP in the striatum was higher in mice injected with HTL‐α‐syn PFFs than these in mice injected with α‐syn PFFs (Figure S7h‐j). The number of tyrosine hydroxylase (TH)‐positive neurons in the SNpc ipsilateral to the injection site was slightly decreased compared with that in mice injected with α‐syn PFFs (Figure S7k,l). Moreover, the loss of TH‐positive nerve terminals in the striatum was also more drastic in mice injected with HTL‐α‐syn PFFs (Figure S7k,m). Western blot analysis found that the levels of TH in the striatum were decreased in mice injected with HTL‐α‐syn PFFs compared with mice injected with α‐syn PFFs (Figure S7n,o). Consistently, the mice injected with HTL‐PFFs showed more severe motor deficits in behavioral tests including the rotarod test, wire hang test, pole test, and balance beam test (Figure S7p‐t). Thus, the homocysteinylated α‐syn PFFs induce more severe α‐syn pathology, loss of dopaminergic neurons, and PD‐like motor impairments.

### 2.7 Elevated levels of brain Hcy exacerbates α‐syn pathology in a mouse model of PD

**FIGURE 5:**
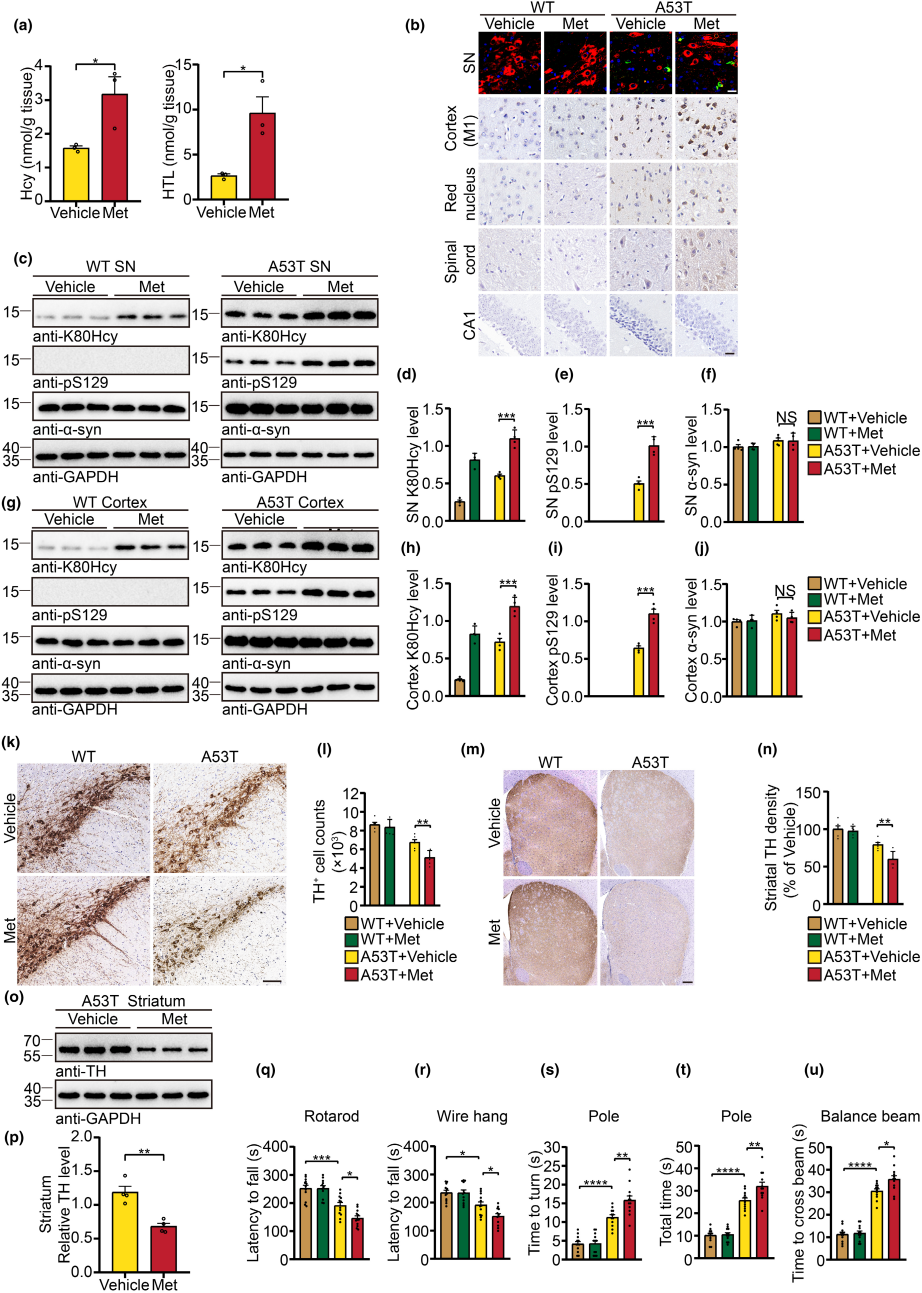
Diet‐induced elevated Hcy worsens α‐syn pathology in TgA53T mice. (a) Brain tissues were assayed by LC–MS for levels of Hcy and GC–MS for levels of HTL. (b) Representative α‐syn K80Hcy (green) and TH (red) double‐immunostaining in the SN, α‐syn K80Hcy immunostaining in the cortex (M1), red nucleus, spinal cord, and hippocampus (CA1). Scale bar is 20 μm. (c–j) Levels of α‐syn K80Hcy, pS129, and human α‐syn levels in the SN (c–f) and cortex (g–j) of TgA53T mice. (k, l) TH immunohistochemistry images in the SNpc. Scale bar is 100 μm. (m, n) TH immunohistochemistry images in the striatum. Scale bar is 200 μm. (o, p) Levels of TH in the striatum. (q–u) Behavioral tests. Shown are the results of rotarod test (q), wire hang test (r), pole test (s, t), and balance beam test (u). *n* = 3 (a), 4 (c–f, g–j, p), 6 (k–n), 12 (q–u) mice per group. All Data are shown as mean ± SEM. *p < 0.05, **p < 0.01, ***p < 0.001, ****p < 0.0001, NS, not significant

We further tested the effect of hyperhomocysteinemia on α‐syn pathology in TgA53T mice. As expected, the levels of Hcy and HTL in the mouse brain were increased after l‐methionine (Met) administration (Figure 5a). The percentage of α‐syn K80Hcy increased from 3.58% in vehicle‐treated TgA53T mice to 6.88% in Met‐treated TgA53T mice (Figure S8a). Six months after the administration of Met, IHC showed that the levels of α‐syn K80Hcy increased in both wild‐type mice and TgA53T mice. The levels of pS129 were higher in Met‐treated TgA53T mice than that in vehicle‐treated TgA53T mice. Met did not trigger the appearance of pS129 in wild‐type mice (Figure S8b). These results were confirmed by Western blot analysis (Figure 5c–j). Met administration also induced an increased IBA1 and GFAP immunoreactivity in TgA53T mice, but not in wild‐type mice (Figure S8c‐j). Furthermore, the number of TH‐positive dopaminergic neurons in the SNpc was decreased in Met‐treated TgA53T mice when compared with vehicle‐treated TgA53T mice (Figure 5k,l). Treatment with Met also induced more severe loss of TH‐positive nerve terminals in the striatum of TgA53T mice (Figure 5m,n). Met treatment did not induce the degeneration of the nigrostriatal pathway in wild‐type mice (Figure 5k–n). The levels of TH were also decreased in the striatum of TgA53T mice treated with Met (Figure 5o,p). Behavioral analysis found that the vehicle‐treated TgA53T mice showed impaired motor function compared with the vehicle‐treated WT mice. The TgA53T mice treated with Met exhibited exacerbated motor deficits in the behavioral tests than the vehicle‐treated TgA53T mice (Figure 5q–u). Thus, elevated levels of Hcy promote α‐syn pathology in vivo.

### 2.8 Blockade of α‐syn K80Hcy ameliorates the toxicity of Hcy in vivo

**FIGURE 6:**
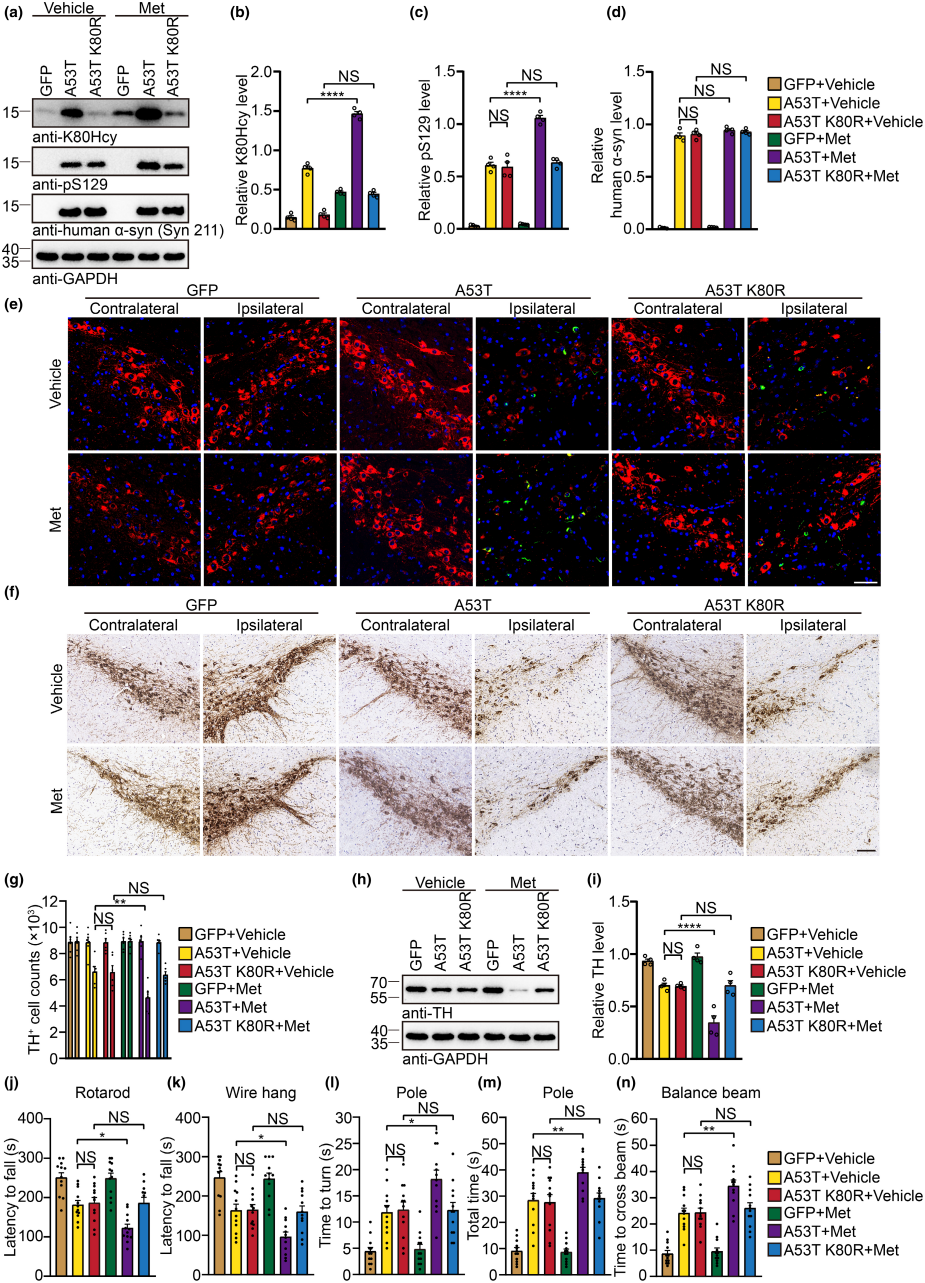
Blockade of α‐syn K80Hcy attenuates α‐syn pathology induced by Hcy. (a–d) Levels of human α‐syn, pS129, and α‐syn K80Hcy in the ipsilateral SN. (e) Representative double‐immunostaining for α‐syn K80Hcy (green) and TH (red) in the SNpc. Scale bar is 50 μm. (f) Representative TH immunohistochemistry images in the SNpc. Scale bar is 100 μm. (g) Stereological counting of the number of TH‐positive neurons. (h, i) Levels of TH in the ipsilateral striatum. (j–n) Behavioral assessment. Shown are the results of the rotarod test (j), wire hang test (k), pole test (l, m), and balance beam test (n). Data are shown as mean ± SEM. *n* = 4 (a–d, i), 6 (g), 12 (j–n) mice per group. *p < 0.05, **p < 0.01, ****p < 0.0001, NS, not significant

AAV‐mediated α‐syn overexpression in the SN has been shown to induce α‐syn pathology in the brain (Oliveras‐Salva et al., 2013). To determine whether α‐syn K80Hcy modification mediates the toxic effect of Hcy, we injected adeno‐associated viruses (AAVs) encoding GFP, human α‐syn A53T, and human α‐syn A53T K80R, respectively, into the SN of three‐month‐old WT mice and fed the mice with vehicle or Met for 6 months. The expression of exogenous α‐syn in the SN was confirmed by immunostaining with an antibody specific to human α‐syn (Syn211) (Figure S9a). Immunofluorescence confirmed that TH‐positive neurons in the SN were efficiently infected by AAVs (Figure S9b). Six months after injection, Western blot showed that the levels of human α‐syn A53T and human α‐syn A53T K80R were comparable. Met treatment enhanced the levels of α‐syn K80Hcy and pS129 in mice injected with AAV‐α‐syn A53T, but not in mice injected with AAV‐α‐syn A53T K80R (Figure 6a–d). The results were further confirmed by immunostaining (Figure 6e and Figure S10a). The deposition of α‐syn K80Hcy and pS129 was accompanied by increased IBA1 and GFAP immunoreactivity (Figure S10b‐e). Histological analysis showed that the overexpression of α‐syn A53T and α‐syn A53T K80R decreased the number of TH‐positive neurons in the SNpc ipsilateral to the injection site. The administration of Met caused a more severe loss of TH‐positive cells in mice injected with AAV‐α‐syn A53T, but not in mice injected with AAV‐α‐syn A53T K80R (Figure 6f,g). Met exacerbated the loss of TH‐positive neuronal terminals and the decrease of TH levels in the striatum in mice expressing α‐syn A53T, but not in mice expressing α‐syn A53T K80R (Figure 6h,i and Figure S10f,g). Consistently, Met promoted behavioral deficits in mice expressing α‐syn A53T, but not in mice expressing α‐syn A53T K80R (Figure 6j–n). Collectively, inhibition of α‐syn K80Hcy antagonizes the detrimental effect of Hcy, supporting the role of α‐syn K80Hcy in the development of α‐syn pathology.

## 3 DISCUSSION

In the current study, we report that N‐homocysteinylation of α‐syn on the K80 residue triggers α‐syn pathology. The levels of α‐syn K80Hcy are increased in an age‐dependent manner, correlating with elevated Hcy and HTL in the brain during aging. α‐Syn K80Hcy is more prone to aggregate and form fibrils with enhanced seeding activity and neurotoxicity both in vitro and in vivo. Chronic exposure to higher Hcy levels promoted the deposition of α‐syn K80Hcy in the brain and induced dopaminergic neuronal degeneration and behavioral deficits in the TgA53T mouse model of PD. Blockade of α‐syn homocysteinylation by mutating the K80 residue to R suppressed α‐syn pathology and behavioral deficits induced by Hcy. These results strongly support the role of N‐homocysteinylation on the onset and progression of α‐syn pathology.

Abnormal Hcy metabolism is a well‐known condition linked to a higher risk of several neurological diseases including stroke, AD, and multiple sclerosis (Chen et al., 2017; Wang et al., 2019). Recent epidemiological evidence indicates that elevated Hcy level is a metabolic risk factor for PD independent of other confounders (Licking et al., 2017; Sapkota et al., 2014). Subjects with serum Hcy levels higher than 20 μmol/L show an 8.64‐fold increased chance of having PD (Saadat et al., 2018). However, the molecular mechanisms underlying the detrimental effect of Hcy remain unclear. Hcy is converted to HTL in error‐editing reactions catalyzed by MARS (Jakubowski, 2011). HTL has been reported to form isopeptide bonds with protein lysine residues, which is known as N‐homocysteinylation (Jakubowski, 1999). N‐homocysteinylation is an emerging posttranslational modification that impairs the protein's structure/function (Jakubowski, 2019).

Genetic or nutritional deficiencies in one‐carbon and Hcy metabolism cause the accumulation of protein N‐homocysteinylation. In the present study, we first used a biorthogonal azide probe to confirm that α‐syn can be homocysteinylated (Figure 1). LC–MS/MS together with point mutations identified that K80 is the major homocysteinylation site of α‐syn. The presence of K80Hcy in the brain of PD mouse models was validated by a polyclonal antibody against α‐syn K80Hcy (Figure 3). K80 resides in the NAC region, which is critical for α‐syn fibrillization and LB formation. Consistently, we found that homocysteinylation of α‐syn promotes its fibrillization, while K80R mutation that blocks homocysteinylation on K80 abolishes the effect of HTL on α‐syn fibrillization in vitro and in vivo. These data strongly support that α‐syn homocysteinylation on the K80 residue facilitates its aggregation, S129 phosphorylation, and neurotoxicity. It is very interesting that the K80 is the major residue modified by N‐homocysteinylation when α‐syn contains various lysine residues. The surrounding amino acids may affect the selectivity of N‐homocysteinylation. Even though N‐homocysteinylation has been reported in many proteins (Bossenmeyer‐Pourié et al., 2019; Zhang et al., 2018), it is hard to predict the exact modification sites until now. No specific enzyme has been reported to mediate the modification of lysine residues.

PD is a heterogeneous disease with many different subtypes. Different patients show distinct patterns of progression, outcomes, and symptoms. Increased levels of Hcy are associated with more severe motor impairment, depression, and cognitive dysfunction in PD patients (O'Suilleabhain et al., 2004). Many factors may contribute to the heterogeneity of PD (Armstrong & Okun, 2020). We found that the homocysteinylated α‐syn fibrils are more potent to seed the aggregation of α‐syn in α‐syn‐HEK293 cells, primary neurons, and mouse brains. Furthermore, we found that increasing the levels of Hcy and HTL in the brain of TgA53T mice enhanced the deposition of α‐syn K80Hcy and pS129, and exacerbated PD‐like motor impairments. This is consistent with the clinical observation that higher Hcy levels are associated with worse motor and non‐motor symptoms of PD (Bakeberg et al., 2019; Christine et al., 2018). Here, we found more severe α‐syn pathology and neurodegeneration in SN of TgA53T mice when compared with previous reports. This might be ascribed to the fact that the background of M83 mice in our study is different from that in the previous reports. The mice in our study were on B6 background, but not B6:C3 background. Thus, the different backgrounds of M83 mice may contribute to the difference in the degree of dopaminergic degeneration.

In patients with elevated Hcy due to mutations in the cystathionine β‐synthase (CBS) gene, HTL concentration also increases 72 folds above its reference value (Chwatko et al., 2007). Increased HTL induces N‐homocysteinylation of the substrate proteins, which leads to a variety of diseases (Bossenmeyer‐Pourié et al., 2019; Wang et al., 2018). Many studies found that target proteins are subjected to extensive N‐homocysteinylation of lysine residues, the only known target of HTL reaction in proteins (Jakubowski, 1999, 2000). Protein N‐homocysteinylation results in the formation of new free thiols. These thiols are prone to oxidation with the consequent production of disulfide bridges, leading to protein oligomerization. This may explain how the homocysteinylation of α‐syn promotes its aggregation. Several types of posttranslational modifications contribute to pathological protein aggregation (Levine et al., 2019; Vicente Miranda et al., 2017). Based on the results reported in this work, we suggest that protein N‐homocysteinylation should also be considered a risk factor for protein conformational diseases including PD.

In conclusion, we present comprehensive evidence that α‐syn homocysteinylation is a mechanism by which Hcy regulates the onset and progression of α‐syn pathology. α‐Syn homocysteinylation contributes to PD‐related conformational change of α‐syn, fibril formation, and neurotoxicity. Thus, monitoring Hcy and homocysteinylated α‐syn levels may represent a promising biomarker for the recognition and diagnosis of PD. Moreover, the discovery of α‐syn homocysteinylation in PD provides a framework for therapeutic intervention.

## 4 MATERIALS AND METHODS

### 4.1 Animals

Adult C57BL/6J mice, *Snca*‐knockout mice, and human A53T variant α‐syn transgenic line M83 were from the Jackson Laboratory (stock number: 000664, 003692 and 004479, respectively). M83 mice were backcrossed with C57BL/6J mice for at least 10 generations to obtain TgA53T mice with C57BL6/J congenic strain. Then the heterozygous male and female TgA53T mice were bred to obtain homozygous offspring and wild‐type littermates. The genotypes were identified using RT‐PCR. Animal maintenance and experiments were performed in accordance with the Declaration of Helsinki and guidelines of Renmin Hospital of Wuhan University. The protocol was reviewed and approved by the Animal Care and Use Committee of Renmin Hospital of Wuhan University (20210103).

### 4.2 Diet treatments of PD mouse models

4‐month‐old TgA53T mice and their wild‐type littermates were given 0.5% l‐methionine (wt/vol, dissolved in drinking water) for 6 months. The mice in the vehicle group received normal drinking water.

### 4.3 Cell culture and treatment

HEK293 cells were from the American Type Culture Collection (ATCC) and tested for mycoplasma contamination before use. The cells were stably expressed with wild‐type α‐syn with a GFP tag directly fused to the C‐terminal of α‐syn (α‐syn‐HEK293 cells). HEK293 and α‐syn‐HEK293 cells were cultured in Dulbecco's modified Eagle's medium (DMEM) containing 10% fetal bovine serum (FBS) and 100 × Penicillin–Streptomycin. Cell lines were maintained at 37°C and 5% CO_2_. L‐Homocysteine (Hcy) (Sigma, 69453) and HTL (Sigma, H6503) were freshly prepared before use. Hcy or HTL were added to the culture media to reach the final indicated concentration (0.1, 0.5, and 1 mM) after the cells were starved in 1% FBS medium for 24 h.

### 4.4 Human tissue samples

Post‐mortem brain samples were from the frozen brain samples from the Emory Alzheimer's Disease Research Center. PD cases were clinically diagnosed and neuropathologically confirmed. The average age of the control and PD patients was 71.8 (*n* = 6) and 71.2 (*n* = 6), respectively. The average disease duration was 6.8 years. Informed consent was obtained from all subjects. The study was approved by the biospecimen committee at Emory University. For immunofluorescent staining, the brain sections were incubated in 0.1% Sudan Black B (SSB) and 70% ethanol to eliminate the autofluorescence signal.

### 4.5 Plasmid constructs and transfection

Cells were transfected with plasmids encoding WT or point‐mutant α‐syn using polyethyleneimine (PEI). For MARS knockdown, HEK293, and α‐syn‐HEK293 cells were transfected with siRNAs using Lipofectamine 2000 (Invitrogen, 11668019). The siRNA sequences used were as follows: sense: 5'‐CCGCUGGUUUAACAUUUCGUU‐3′, antisense: 5′‐ ACGAAAUGUUAAACCAGCGG‐3′.

### 4.6 Protein purification

cDNAs corresponding to his‐tagged α‐syn and K80R mutant were cloned into a PRK172 plasmid and transformed into the E. coli BL21 DE3 strain. The expression and purification were performed as previously described (Dai et al., 2021). Briefly, the pellet from 1 L culture was resuspended in 100 ml osmotic shock buffer (30 mM Tris–HCl, 40% sucrose, and 2 mM EDTA, pH 7.2) and incubated for 10 min at room temperature. The pellet collected by centrifugation at 12,000 rpm for 20 min was resuspended quickly with 90 ml cold water followed by adding 37.5 μl of saturated MgCl_2_, and kept on ice for 3 min. His‐tagged proteins were purified through Ni‐chelating affinity chromatography and eluted at around 125 mM imidazole. The protein was dialyzed, lyophilized, and stored at −80°C before use.

### 4.7 Preparation and transduction of α‐syn fibrils

PFFs were generated by incubating purified protein (1 mg/ml) at 37°C with constant agitation (1000 rpm) for 5–7 days. 1 mg/ml WT or K80R mutant α‐syn was mixed with vehicle or HTL at 37°C, rotated at 250 rpm for 8 h, and then dialyzed against PBS. The samples were shaken at 37°C at 1000 rpm for 5–7 days in an Eppendorf thermomixer C and monitored by ThT fluorescence at various time points. Briefly, aliquots of 10 μl incubation samples were diluted to 100 μl with 20 μM ThT in PBS and tested at 450 nm excitation and 510 nm emission using Spectra Max plate reader (Molecular Devices). The fibrils were collected by centrifugation at 100,000 *g* for 20 min and resuspended in sterile PBS. Before transduction, the fibrils were sonicated with 60 pulses at 10% power (total of 30, 0.5 sec on, 0.5 sec off), and then quantified using an ELISA kit. α‐Syn fibrils (140 ng/ml, final concentration) were transduced using Lipofectamine 2000. HTL was added 12 h before transduction with fibrils and incubated during the whole experiment. The cells were then transfected with fibrils and incubated for another 24 h.

### 4.8 Transmission electron microscopy (TEM)

A 20 μl droplet from each sample was dropped onto the copper grid with carbon film for 3–5 min. Two percentage of phosphotungstic acid was dropped on the copper grid to stain for 1–2 min. Grids were allowed to dry at room temperature. Images were obtained using TEM.

### 4.9 Proteinase K and pronase digestion

The PFFs samples (19 μl, 19 μg) were mixed with 1 μl of Proteinase K (2.5 μg/ml final concentration) or pronase (50 μg/ml final concentration), and incubated at 37°C for 30, 60, and 90 min, after which, 5 μl of 5× SDS loading buffer were added to quench the reactions, and then boiled for 10 min at 95°C. Afterward, 6 μl of each digested product was separated by SDS‐PAGE. Gels were stained with Coomassie brilliant blue R‐250 (BioFroxx, 1912GR025).

### 4.10 Sequential extraction

Soluble and insoluble cell fractions were prepared as previously described (Volpicelli‐Daley et al., 2014). In brief, cells were scraped into 1% Triton X‐100 (TX‐100) in Tris‐buffered saline (TBS) (50 mM Tris, 150 mM NaCl, pH 7.4) with protease and phosphatase inhibitor cocktail, and incubated on ice for 30 min. Lysates were then sonicated and centrifuged at 100,000 *g* for 30 min. The supernatant was collected as the soluble fraction. The pellet was washed with 1% TX‐100 in TBS, sonicated, and centrifuged at 100,000 *g* for 30 min. The supernatant was discarded. The pellet was suspended in 2% SDS in TBS as the insoluble fraction.

### 4.11 Western blot analysis

Cells were lysed with ice‐cold NP‐40 lysis buffer containing a cocktail of protease inhibitors and phosphatase inhibitors. Dissection of SN from mice was performed as previously described (Salvatore et al., 2012). Tissues were homogenized in ice‐cold RIPA lysis buffer containing protease and phosphatase inhibitors. The lysates were then sonicated briefly and centrifuged at 21,130 *g* for 20 min. The protein concentration was measured using the BCA assay. Proteins extracts were separated by 10% Bis‐Tris SDS‐PAGE gels. The following primary antibodies were used: HA (1:5000, Proteintech, 51064‐2‐AP), GAPDH (1:8000, Proteintech, 60004‐1‐Ig), Streptavidin‐HRP (1:5000, Proteintech, SA00001‐0), MARS (1:10000, Proteintech, 14829‐1‐AP), GST (1:10000, Proteintech, 66001‐2‐Ig), K80Hcy (1:1000, Abmart), α‐Synuclein (D37A6) (1:1000, Cell Signaling Technology, #4179), Syn211 (1:1000, Thermo Fisher Scientific, MA5‐12272), pS129 (1:1000, Cell Signaling Technology, #23706), GFP (1:10,000, Proteintech, 66002‐1‐Ig), TH (1:1000, Millipore, #AB152). The following secondary antibodies conjugated to horseradish peroxidase (HRP) were used: goat anti‐rabbit IgG (H + L)‐HRP (1:8000, Bio‐Rad, 1706515), goat anti‐mouse IgG (H + L)‐HRP (1:8000, Bio‐Rad, 1706516). Signals were developed by detecting enhanced chemiluminescent (ECL) with Imaging System (Bio‐Rad, ChemiDoc™ Touch). Blot intensity was analyzed and quantified using Image J.

### 4.12 Immunohistochemistry

IHC Detection System Kit (ZSGB‐BIO, PV‐6001/PV‐6002) was used. The sections were incubated with primary antibodies against TH (1:2000, Abcam, ab117112), pS129 (1:1000, Biolegend, 825701), IBA1 (1:500, Wako, 019–19741), GFAP (1:500, Thermo Fisher Scientific, PA5‐16291), Syn211 (1:1000, Thermo Fisher Scientific, MA5‐12272), or K80Hcy antibody (1:500, Abmart) at 4°C for overnight. The signal was developed using DAB. The levels of immunoreactivity were determined by optical density analysis using Image J, plus the IHC Profiler plugin.

### 4.13 Immunofluorescence

Neurons were fixed with 4% paraformaldehyde (PFA) and 0.1% TX‐100 in PBS for 15 min. To detect the insoluble α‐syn aggregates, α‐syn‐HEK293 cells were fixed with 4% PFA in PBS followed by permeabilization with 1% TX‐100. Cells were incubated with anti‐pS129 (1:1000, Biolegend, 825701), anti‐Ubiquitin (1:500, Cell Signaling Technology, #3936) overnight at 4°C. The samples were stained with corresponding secondary antibodies Alexa Fluor 594 or 488 (1:1000, Invitrogen). Nuclei were visualized with DAPI (1 μg/ml, BioFroxx, 1155MG010) for 5 min. To quantify the percentage of positive cells, a total of 8 fields, each with 100+ cells, were analyzed per condition. The primary cultured neurons and brain sections were double stained for MAP2/pS129, pS129/K80Hcy, Thioflavin S (ThS)/K80Hcy, TH/pS129, TH/K80Hcy, or GFP/TH.

### 4.14 Primary neuron cultures

Primary cortical neurons dissected from E18 embryos were cultured as previously described (Zhang et al., 2017). PFFs were added to the culture medium at 7 days in vitro (DIV). Seven days later, the neurons were fixed in 4% PFA, permeabilized, and immuno‐stained with MAP2/pS129 antibodies.

### 4.15 Stereological quantification of TH‐positive cells and striatal terminals

The number of TH‐positive cells in the SN was estimated with a random‐sampling stereological counting method. Every sixth section from the caudal to rostral boundaries of the SN was incorporated into the counting procedure. TH‐positive fiber densities in the striatum were measured by optical density analysis using Image J, plus the IHC Profiler plugin.

### 4.16 Behavioral tests

In the rotarod test, mice were placed on an accelerating rotarod cylinder, from 4 rpm up to 40 rpm within 300 s, and the latency time to fall off was documented. In the pole test, the pole was made up of a 75 cm long wooden rod that was wrapped with bandage gauze. Mice were placed on the top of the pole facing the head‐up. The time taken to orient downward and the total time taken to reach the base were recorded. In the wire hang test, the mice were placed on a horizontal wire grid. The wire was lightly shaken to make the mice grab the wire and then turned upside down. The latency of mice to fall off was measured. Trials were stopped if the mice remained on the grid for 5 min. In the balance beam test, the mice walked along a narrow beam suspended between a start platform and a dark resting box. The time to cross the beam (2 × 100 cm) was recorded.

### 4.17 Stereotaxic injection of α‐syn PFFs

Two‐month‐old C57BL/6J mice were anesthetized and stereotaxically injected in one hemisphere with sonicated fibrils (5 μg each mouse) at the following coordinates: anteroposterior (AP) +0.2 mm; mediolateral (ML) −2.0 mm; dorsoventral (DV) −2.7 mm relative to Bregma. The inoculums were performed using a 10 μl Hamilton syringe at a rate of 200 nl per min with the needle in place for 5 min. Animals were monitored regularly following recovery from surgery.

### 4.18 Viral construction and stereotaxic injection

AAV particles encoding human WT and K80R mutant α‐syn with the human synapsin I (hSyn I) promoter (Kügler et al., 2001) were prepared by BrainVTA (BrainVTA Co., Ltd.). Unilateral intracerebral injection of AAVs was performed stereotaxically at coordinates anteroposterior (AP) −3.1 mm and mediolateral (ML) −1.2 mm relative to the bregma, and dorsoventral (DV) −4.0 mm from the dural surface in three‐month‐old C57BL/6J mice. A total of 300 nl of viral suspension was injected into each site with a 10 μl glass syringe with a fixed needle at a rate of 40 nl/min. The needle remained in place for an additional 5 min before it was removed slowly.

### 4.19 Mass spectrometry analysis

The LC–MS/MS identification and data analysis were performed by SpecAlly Life Technology (SpecAlly Life Technology Co., Ltd.). The target protein bands in the gel were cut into pieces, washed three times with 50% acetonitrile/100 mM NH_4_HCO_3_, and digested in 50 mM NH_4_HCO_3_ solution (pH 8.0) with MS‐grade trypsin overnight at 37°C after reduction and alkylation of cysteines. The tryptic digests were injected into an Easy‐nLC 1200 system (Thermo Scientific) and analyzed by a Q Exactive plus mass spectrometer (Thermo Scientific). Peptides were first loaded onto a C18 trap column (Thermo, 75 μm × 2 cm, 3 μm particle size, 100 Å pore size) and then separated in a C18 analytical column (Thermo, 75 μm × 250 mm, 2 μm particle size, 100 Å pore size). Mobile phase A (0.1% formic acid) and mobile phase B (80% acetonitrile, 0.1% formic acid) were used to establish the separation gradient. A constant flow rate was set at 300 nl/min. For the data‐dependent acquisition (DDA) mode analysis, each scan cycle was consisted of one full‐scan mass spectrum (R = 70 K, AGC = 3E6, max IT = 50 ms, scan range = 350–1800 m/z) followed by 15 MS/MS events (R = 15 K, AGC = 1E5, max IT = 50 ms). The collision energy of high energy capture dissociation (HCD) was set to 27. The isolation window for precursor selection was set to 1.6 Da. Former target ion exclusion was set for 45 s. The raw files were analyzed with Proteome Discoverer (version 2.4) using the Sequest HT search algorithm. Spectra files were searched against the human Uniprot Proteome FASTA database using the following parameters: type, identification; variable modifications: oxidation of methionine (Met), protein N‐terminal acetylation, and N‐homocysteinylation of lysine (KHcy, +174.04600 Da, lysine) (Zhang et al., 2018); fixed modification: carbamidomethyl of cysteine (Cys); digestion, trypsin. The MS1 match tolerance was set as 10 ppm; the MS2 tolerance was set as 0.02 Da. Minimal peptide length was set to six amino acids, and a maximum of three miscleavages was allowed. Search results were filtered with 1% FDR at both protein and peptide levels.

### 4.20 Generation of K80Hcy antibody

To generate the K80Hcy antibody, the synthesized peptide TAVAQKTVEG containing K80 homocysteinylation was used as an antigen to immunize rabbits. The antibody was produced by Abmart Shanghai Co., Ltd. Antiserum was collected after five sessions of immunization. The titers against the immunizing peptide were determined by ELISA.

### 4.21 Detection of Hcy and HTL levels in the brain tissue

All detections were performed by Sensichip Biotech Company (Sensichip Biotech Co., Ltd.). For Hcy level detection, brain tissues were added with 20 μl of 500 mM DTT, 380 μl of extraction solution (40:40:20 acetonitrile: methanol: water). All the samples were vortexed, ground, incubated at room temperature for 30 min, sonicated for 10 min at 4°C, and then centrifuged at 13,523 *g*, 4°C for 10 min. Then, the supernatants were evaporated, reconstituted with 150 μl of 60:40 acetonitrile: water, vortexed well, and clarified by centrifugation at 13,523 *g* for 10 min at 4°C. The supernatants were transferred to an injection vial for liquid chromatography‐mass spectrometry (LC–MS) analysis. The LC–MS/MS method involved Waters Acquity ultraperformance LC (UPLC) coupled to the AB Sciex 5500 QQQ‐MS. The LC separation was performed using an Acquity UPLC BEH Amide (1.7 μm, 2.1 mm × 100 mm). Solvent A was 90:10 water: acetonitrile with 10 mM ammonium acetate and 0.2% acetic acid, and solvent B was 10:90 water: acetonitrile with 10 mM ammonium acetate and 0.2% acetic acid. The flow rate was 0.30 ml/min. The column temperature was 40°C. The injection volume was 10 μl. MS parameters were as follows: curtain gas, 35 arb (arbitrary units); collision gas, 9 arb; IonSpray voltage, 4500 V; IonSource temperature, 450°C; IonSource gas1, 55 arb; and IonSource gas2, 55 arb. According to the conditions described above, the prepared standard Hcy solution was added to the sample vial to quantify and identify the Hcy peaks at Rt = 2.52 min.

The concentration of HTL was performed as previously described (Piechocka et al., 2020) by using Thermo Trace 1300 gas chromatography system coupled with ISQ7000 mass spectrometry (GC–MS). Briefly, brain samples were mixed with 100 μl of 0.2 mol/L phosphate buffer (pH 7.8). The mixtures were extracted by grinding for 5 min with 800 μl of 2:1 chloroform: methanol (v/v). After centrifugation (12,000 rpm, 4°C, 5 min), the organic layer was transferred and dried under a vacuum. The residues were derivatized with 30 μl MSTFA (with 1% TMCS, 50°C, 10 min) for samples. After cooling to room temperature, reaction mixtures were transferred to HPLC vials and an aliquot (1 μl) was injected into the GC–MS system. The capillary column was DB‐5MS (60 m × 0.25 mm × 0.25 μm). The instrument parameter settings were as follows: Inlet temperature, 280°C; EI temperature, 230°C; carrier gas, helium (purity > 99.999%); splitless injection, 1 μl sample. Temperature program: maintain at 146°C for 5 min, ramp from 146°C to 200°C at 5°C/min, the ramp from 200°C to 300°C at 50°C/min, then hold at 300°C for 3 min. Scanning from 50 to 500 (m/z). The MS detector was focused on the trimethylsilyl‐HTL derivative ions with m/z 73.0, m/z 100.0, and m/z 128.0, while m/z 100.0 was selected for quantification. All values multiplied volume and were converted to that relative to weight by dividing tissue weights.

### 4.22 ELISA for K80Hcy quantification

We developed an ELISA‐based method for quantifying the K80Hcy modification. Nunc MaxiSorp plates were coated with K80Hcy antibodies and incubated overnight at 4°C. The plates were washed with washing buffer, and blocked using blocking buffer at room temperature for 1 h. Then, the plates were incubated with the samples at 4°C overnight. The plates were then washed and biotin‐labeled anti‐α‐syn (Biolegend, 807808) was added and incubated at 4°C overnight. After washing, avidin‐HRP (Biolegend, 405103) was added and incubated for 2 h at room temperature. Lastly, the plates were developed using 1‐Step Ultra TMB‐ELISA substrate solution (Thermo Fisher Scientific, 34028) for 15 min. The reaction was quenched with stop solution and plates were read at 450 nm. A standard was generated using K80Hcy‐modified α‐syn, which was prepared in vitro by incubating 60 mM HTL and 1 mg/ml α‐syn overnight. MS analysis showed that 86.27% of α‐syn was modified at K80 in the standard. Thus, this ELISA system slightly overestimates the degree of modification.

### 4.23 Chemoselective labeling of N‐homocysteinylated α‐syn

The reactions were performed as previously reported (Chen et al., 2019). Reaction solutions were labeled by Biotin‐azide (200 μM, final concentration, MedChemExpress, HY‐129832). Freshly made hemin (50 μM, final concentration), β‐Mercaptoethanol (100 mM, final concentration), and SDS (0.4%, final concentration) were added together. The mixtures were heated at 75°C for 10 min. Then, 5× loading buffer was added, and the samples were heated at 95°C for 10 min, followed by 10% Bis‐Tris SDS‐PAGE.

### 4.24 Statistical analysis

All data were shown as mean ± standard error (SEM) of the mean if not mentioned otherwise. Statistical analysis was performed using either an independent sample *t* test (two‐group comparison) or one‐way ANOVA among more than two groups followed by Tukey's post hoc test using GraphPad Prism 8.0.1 (GraphPad Software Inc.). Normality of the data was tested with the Shapiro–Wilk test. For the data that was non‐normally distributed, Mann–Whitney U test (two groups), and Kruskal–Wallis test with Dunn's multiple comparisons (three or more groups) were applied. Statistical tests were two‐tailed, and differences with *p* < 0.05 were considered statistically significant.

## AUTHOR CONTRIBUTIONS

Z.Z. and B.A. conceived the project. Z.L. performed most of the experiments, analyzed the data, and wrote the manuscript. G.T. performed some of the in vitro experiments. M.L., Z.X., T.Y., and D.L. helped in designing the methodology. L.Y. helped with cell culture. L.C. helped in behavioral tests. N.X., C.G., L.C., K.W., and Z.Z. helped with the animal experiments. Z.Z. supervised the entire project.

## FUNDING INFORMATION

This work was supported by grants from the National Key Research and Development Program of China (2019YFE0115900) to Z.Z., the National Natural Science Foundation of China (No. 82271447 to Z.Z., No. 81901090 to L.M), and Medical Science Advancement Program of Wuhan University (No. TFLC2018001).

## CONFLICT OF INTEREST

The authors declare no competing interests.

## Supporting information


**Appendix S1:** Supporting Information

## Data Availability

The authors declare that all data supporting the findings of this study are available within the article and its supplementary information files.
