# Supplementary material for: N‐homocysteinylation of α‐synuclein promotes its aggregation and neurotoxicity
Source: Aging Cell. 2022 Nov 27;22(3):e13745. doi: 10.1111/acel.13745 (PMC10014048; doi:10.1111/acel.13745)
Supplement: Supplementary file 1 — Appendix S1: Supporting Information [file ACEL-22-e13745-s001.docx]

**Supplementary Information**


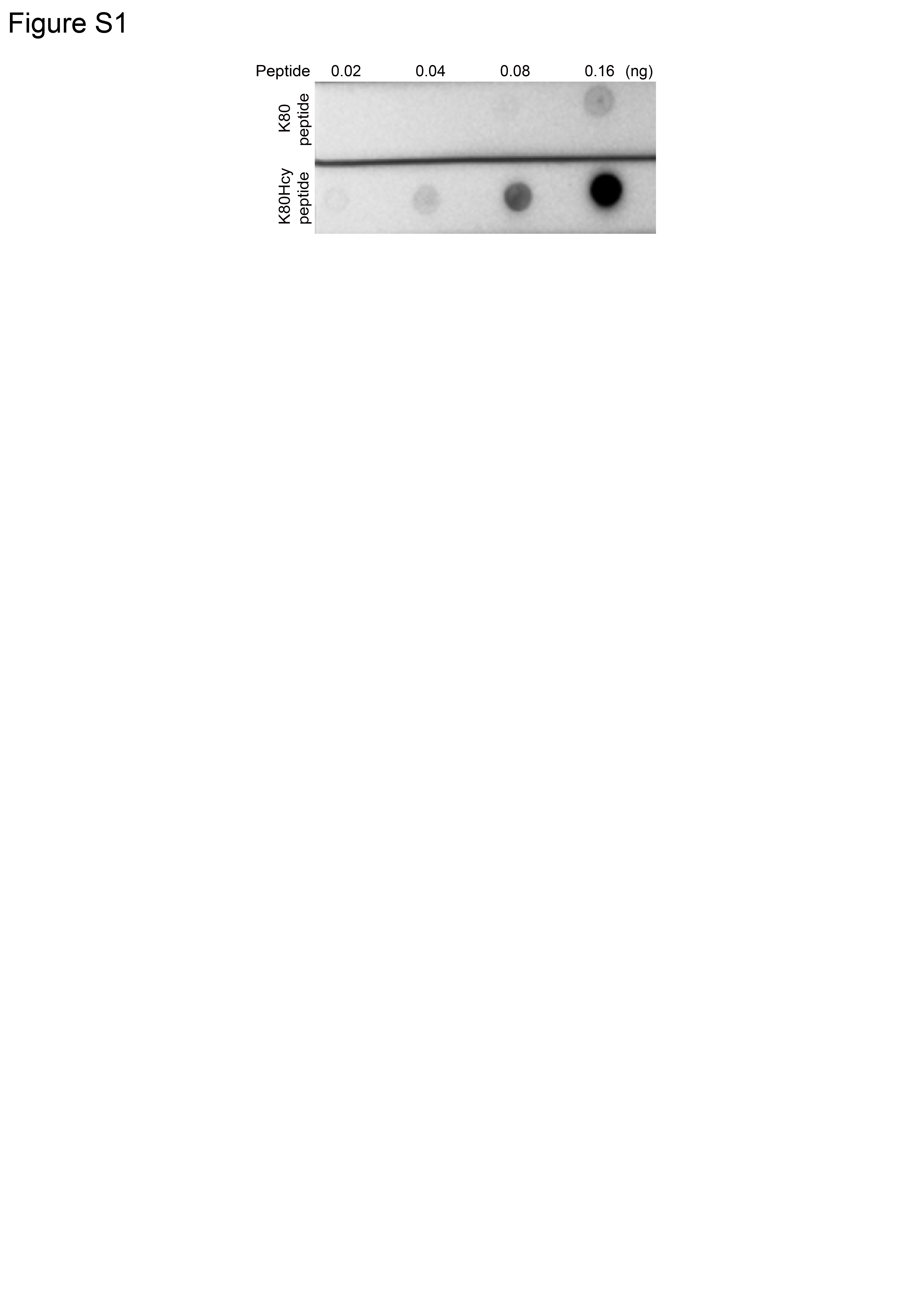


**Figure S1. Verification of the antiserum against α-syn K80Hcy.**

Dot blot analysis using non-homocysteinylated and homocysteinylated K80 peptide were used to confirm the reactivity of the α-syn K80Hcy antiserum.


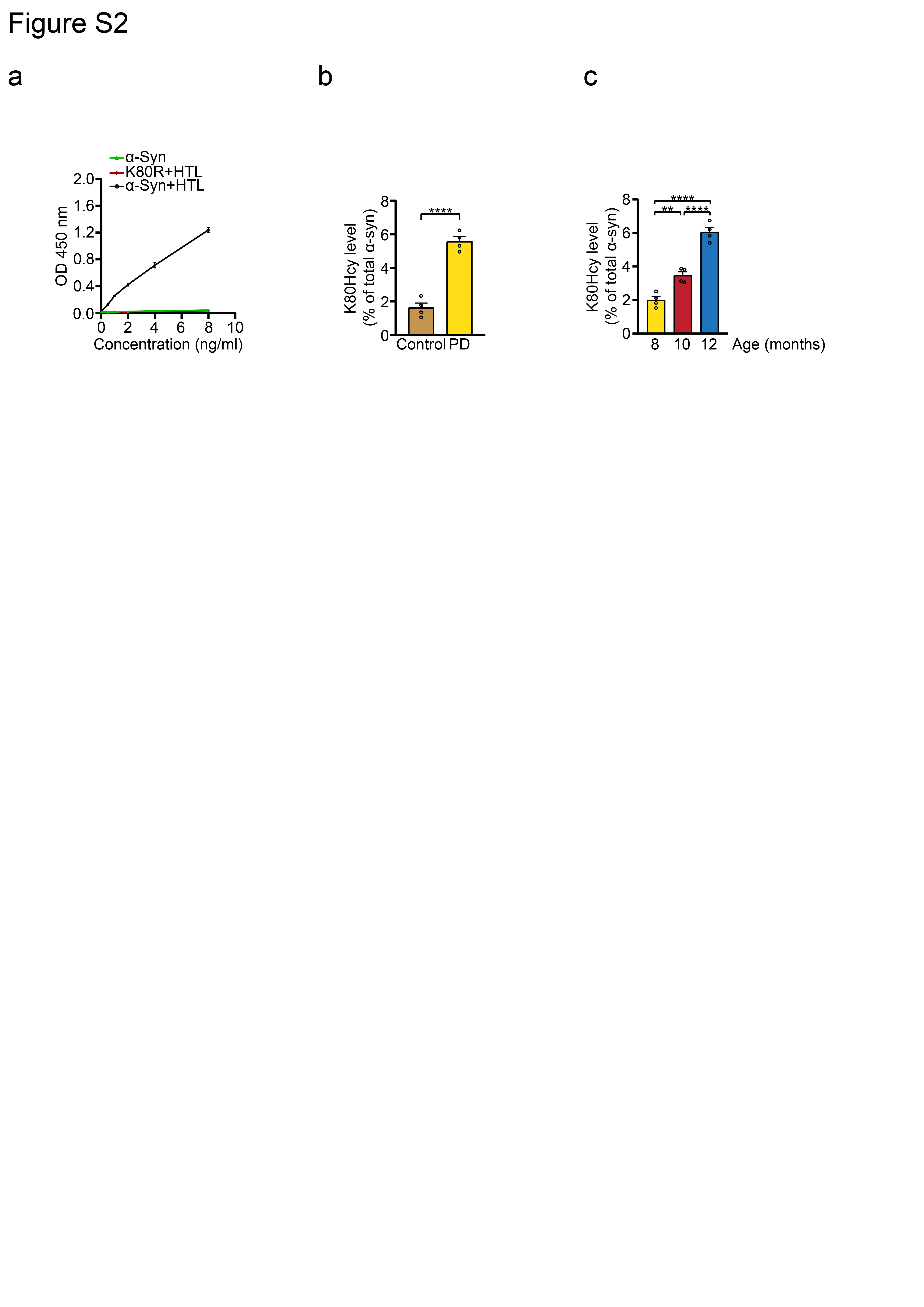


**Figure S2.** **Quantitation of α-syn K80Hcy.**

(**a**) OD 450 in ELISA analysis using anti-K80Hcy antibody. The ELISA kit recognizes HTL-treated WT α-syn, but not HTL-treated K80R mutant α-syn. (**b**) The percentage of α-syn K80Hcy in the brain of control subjects and PD patients. (**c**) The percentage of α-syn K80Hcy in the TgA53T mice at different ages. Data are shown as mean ± SEM. n = 4 independent experiments. **P < 0.01, ****P < 0.0001.


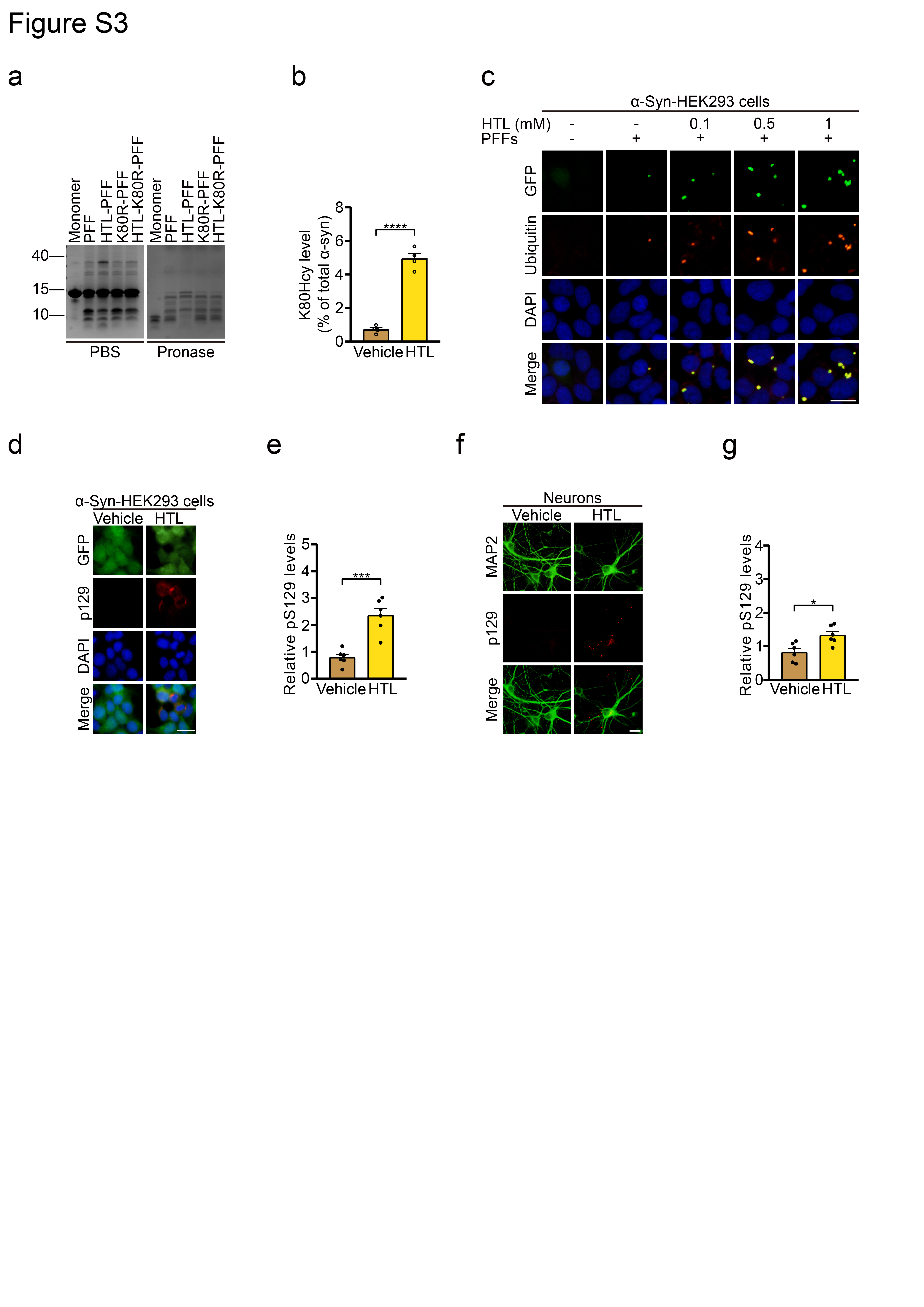


**Figure S3. HTL promotes α-syn aggregation.**

(**a**) Pronase digestion of different fibrils. Fibrils (19 μL, 19 μg) were digested with pronase (50 μg/mL final concentration) at 37 °C for 60 min. (**b**) The percentage of α-syn K80Hcy in vehicle- and HTL-treated α-syn-HEK293 cells. (**c**) Representative images showing the co-localization of aggregated α-syn with ubiquitin in α-syn PFFs-treated α-syn-HEK293 cells in the presence or absence of HTL. (**d,e**) α-Syn-HEK293 cells were exposed to 0.1 mM HTL. Immunofluorescence shows α-syn phosphorylation at S129. Scale bar is 20 μm. (**f,g**) Neurons were exposed to 0.1 mM HTL. Immunofluorescence shows α-syn phosphorylation at S129. Scale bar is 20 μm. Data are shown as mean ± SEM. n = 4 (b), 6 (e,g) independent experiments. *P < 0.05, ***P < 0.001, ****P < 0.0001.


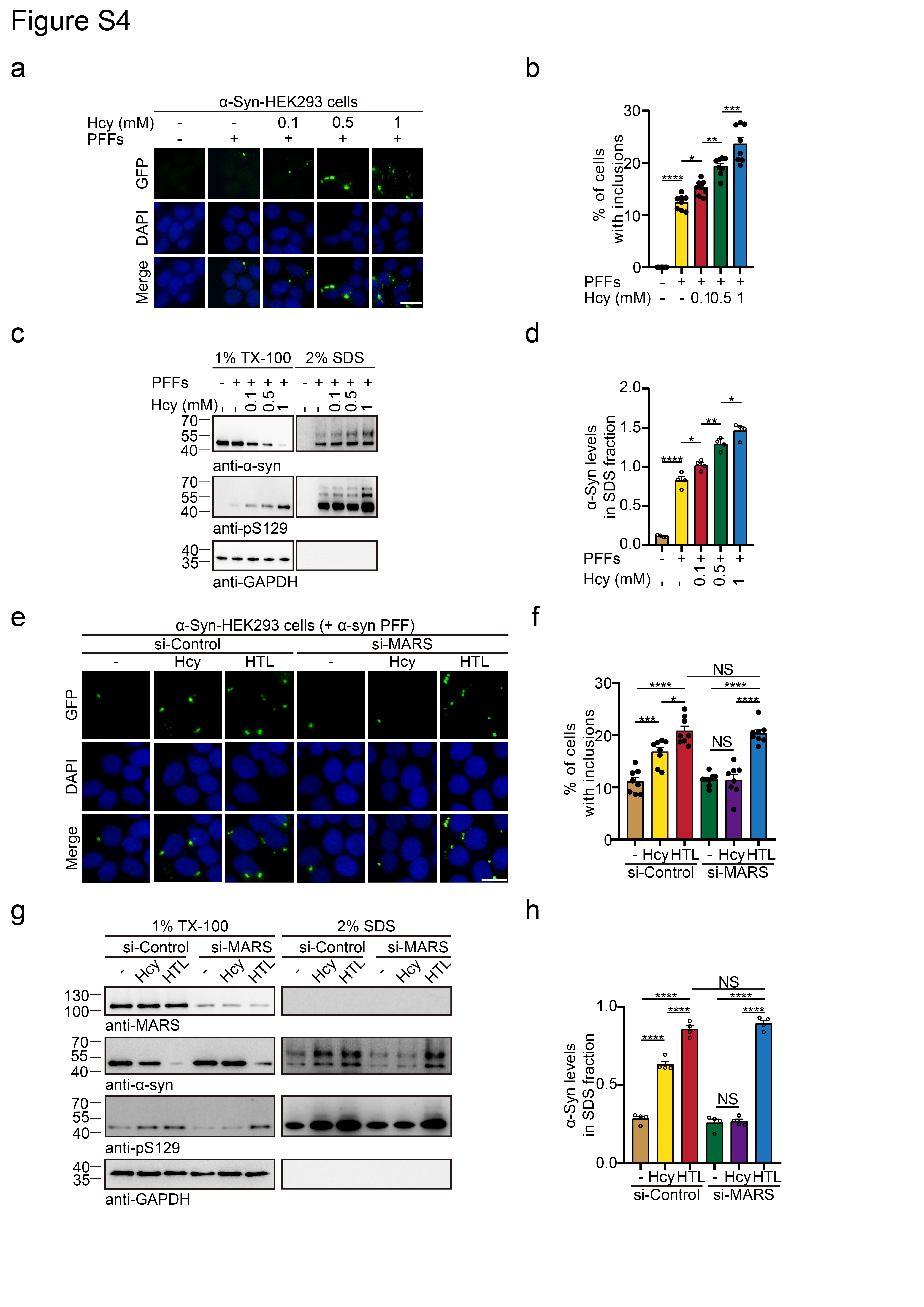


**Figure S4. Hcy promotes α-syn aggregation, which is dependent on MARS.**

(**a-d**) The α-syn-HEK293 cells were exposed to Hcy, and then transduced with α-syn PFFs. (**a,b**) Images and quantification of insoluble α-syn inclusions. (**c,d**) Western blot analysis of Syn211 and pS129 in Triton X-100-soluble and SDS-soluble fractions. (**e-h**) α-Syn-HEK293 cells were transfected with MARS siRNA or control siRNA, treated with Hcy (0.1mM) or HTL (0.1mM), and then transduced with α-syn PFFs. (**e,f**) Images and quantification of insoluble α-syn inclusions. (**g,h**) Western blot analysis of Syn211 and pS129 in Triton X-100-soluble and SDS-soluble fractions. n = 8 (b,f), 4 (d,h) independent experiments. All data are shown as mean ± SEM. *P < 0.05, **P < 0.01, ***P < 0.001, ****P < 0.0001, ^NS^ not significant. Scale bar is 20 μm.


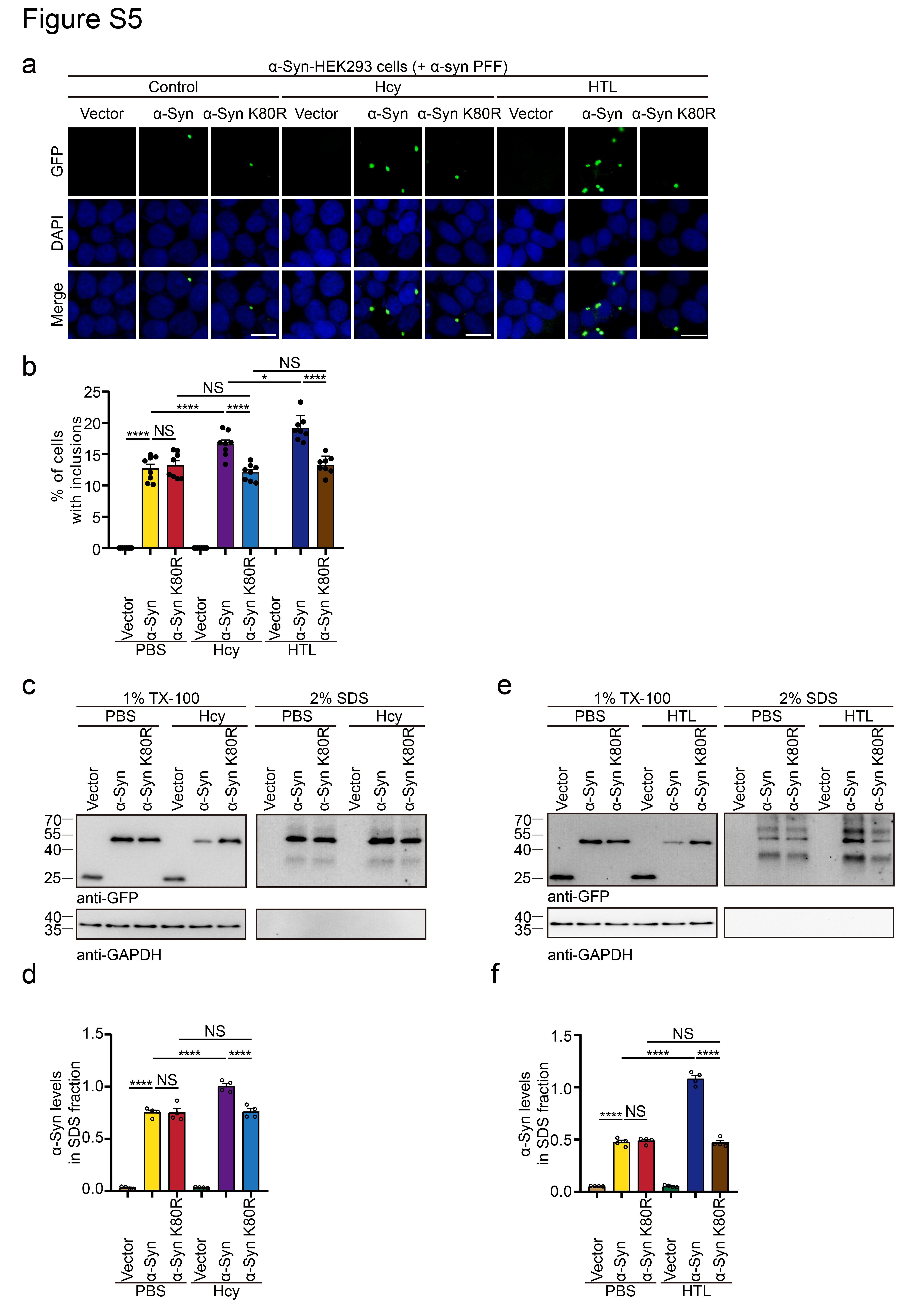


**Figure S5.** **K80R mutation abolishes the effect of Hcy on α-syn aggregation.**

(**a,b**) HEK293 cells were transfected with wild-type or K80R mutant α-syn, treated with Hcy (0.1mM) or HTL (0.1mM), and then transduced with α-syn PFFs. (**a**) The green dots show α-syn inclusions. (**b**) Quantification of the percentage of cells with α-syn inclusions. (**c-f**) Western blot analysis of GFP in Triton X-100-soluble and SDS-soluble fractions. n = 8 (b), 4 (d,f) independent experiments. All data are shown as mean ± SEM. *P < 0.05, ****P < 0.0001, ^NS^ not significant. Scale bar is 20 μm.


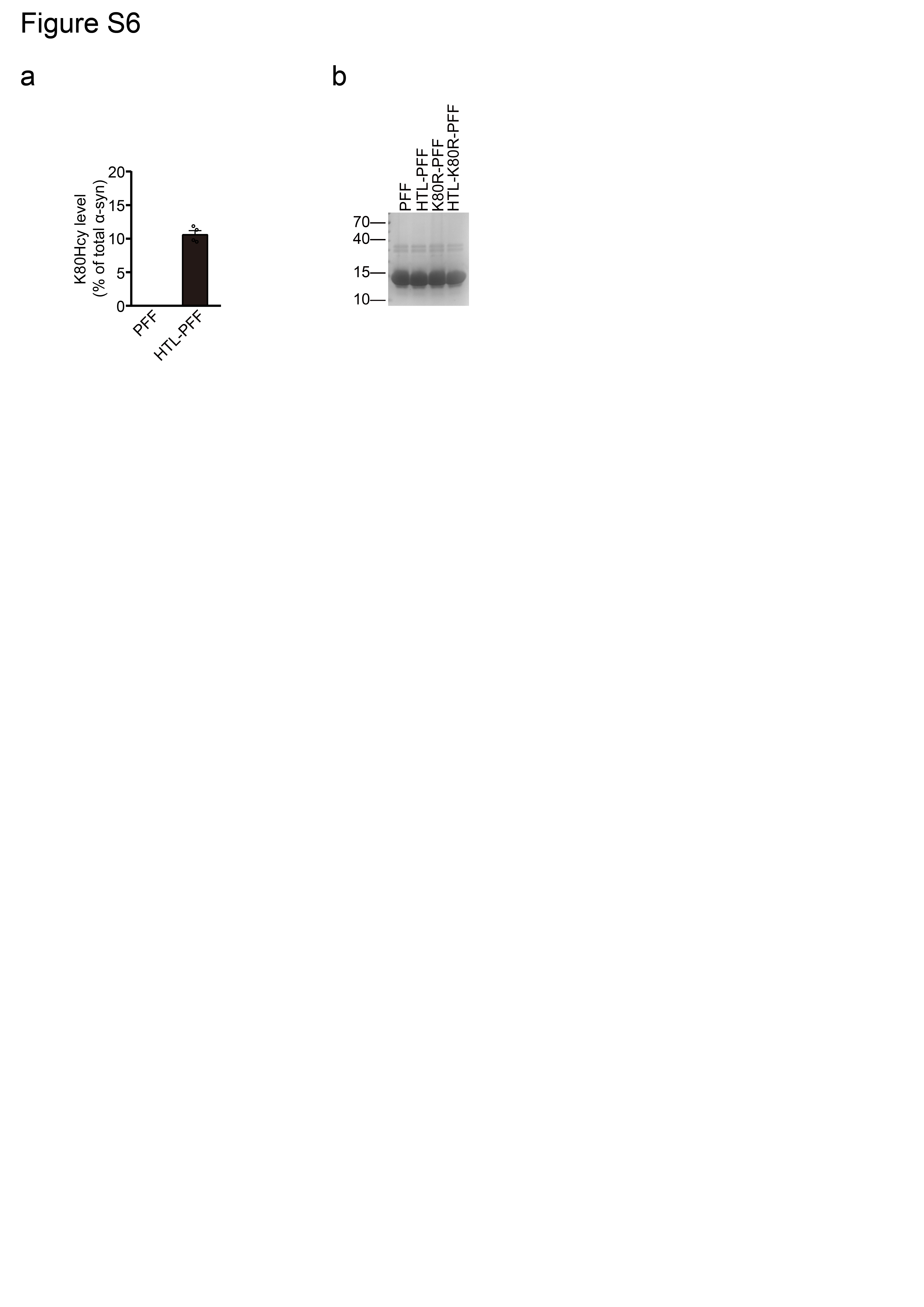


**Figure S6.** **The characteristics of α-syn PFFs.**

(**a**) The ratio of K80 modification in α-syn PFFs. (**b**) Coomassie Blue stained gel showing the same amounts of fibrils were used in the experiments.


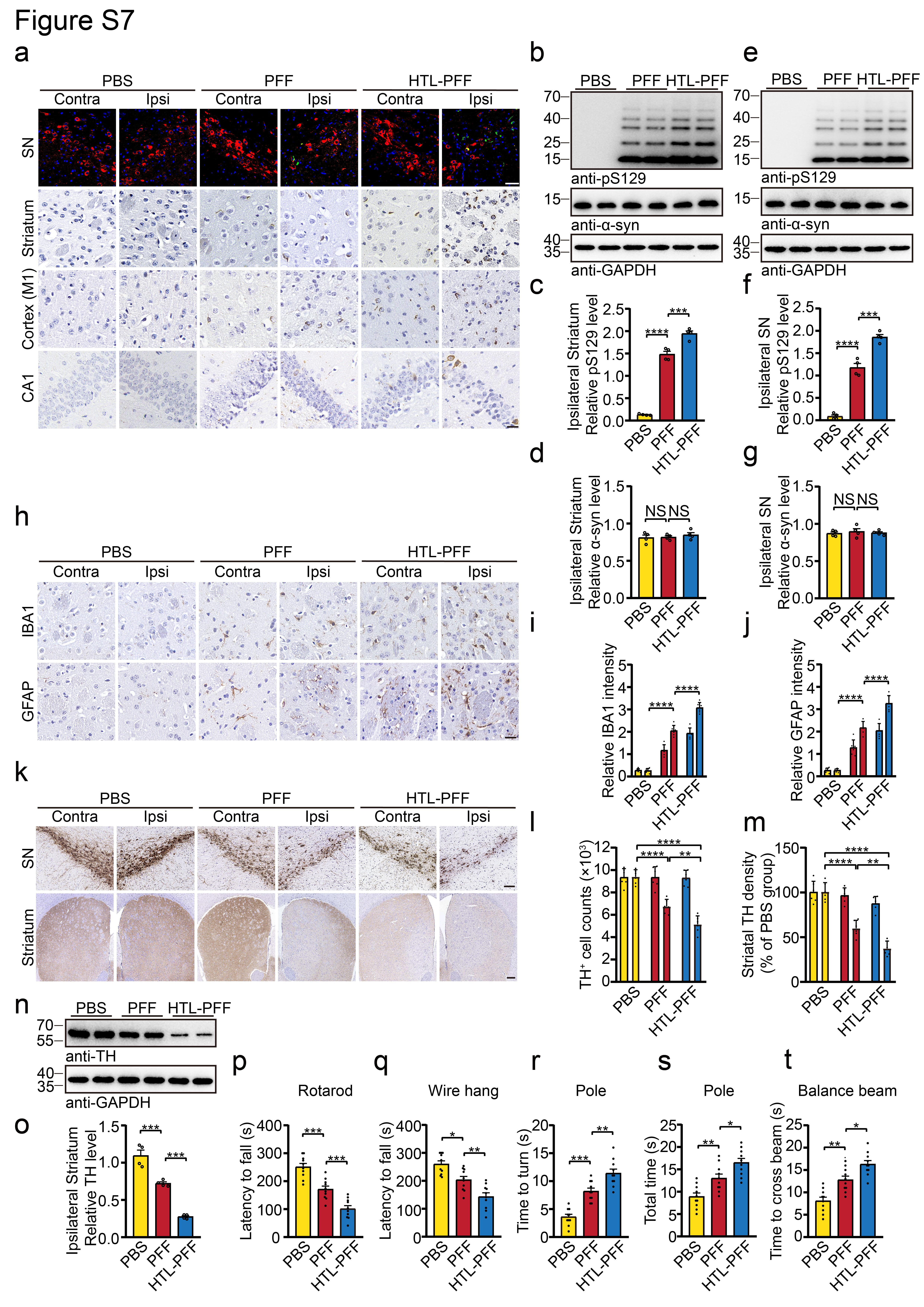
**Figure S7. HTL-modified α-syn PFFs are more neurotoxic *in vivo*.**

(**a**) Representative pS129 (green) and TH (red) double-immunostaining in the SN, pS129 immunostaining in the striatum, cortex (M1), and hippocampus (CA1) of WT mice. Scale bar is 50μm (SN) or 20 μm (striatum, M1, and CA1). Western blots of pS129 and α-syn in the ipsilateral striatum (**b-d**) and SN (**e-g**) of WT mice. (**h**) Representative IBA1 and GFAP immunostaining in the striatum. Scale bar is 20 μm. (**i,j**) Intensity of IBA1 and GFAP positive signals. (**k**) Representative TH immunohistochemistry images in the SNpc and striatum of WT mice. Scale bar is 100μm (SN) or 200 μm (striatum). (**l**) The number of TH-positive neurons in the SNpc of WT mice. (**m**) Density of TH-positive terminals in the striatum of WT mice. (**n,o**) Levels of TH in the ipsilateral striatum of WT mice. (**p-t**) Behavioral tests including the rotarod test (**p**), wire hang test (**q**), pole test (**r, s**), and balance beam test (**t**). n = 4 (b-g,o), 6 (h-m), 10 (p-t) mice per group. All data are shown as mean ± SEM. *P < 0.05, **P < 0.01, ***P < 0.001, ****P < 0.0001, ^NS^ not significant.


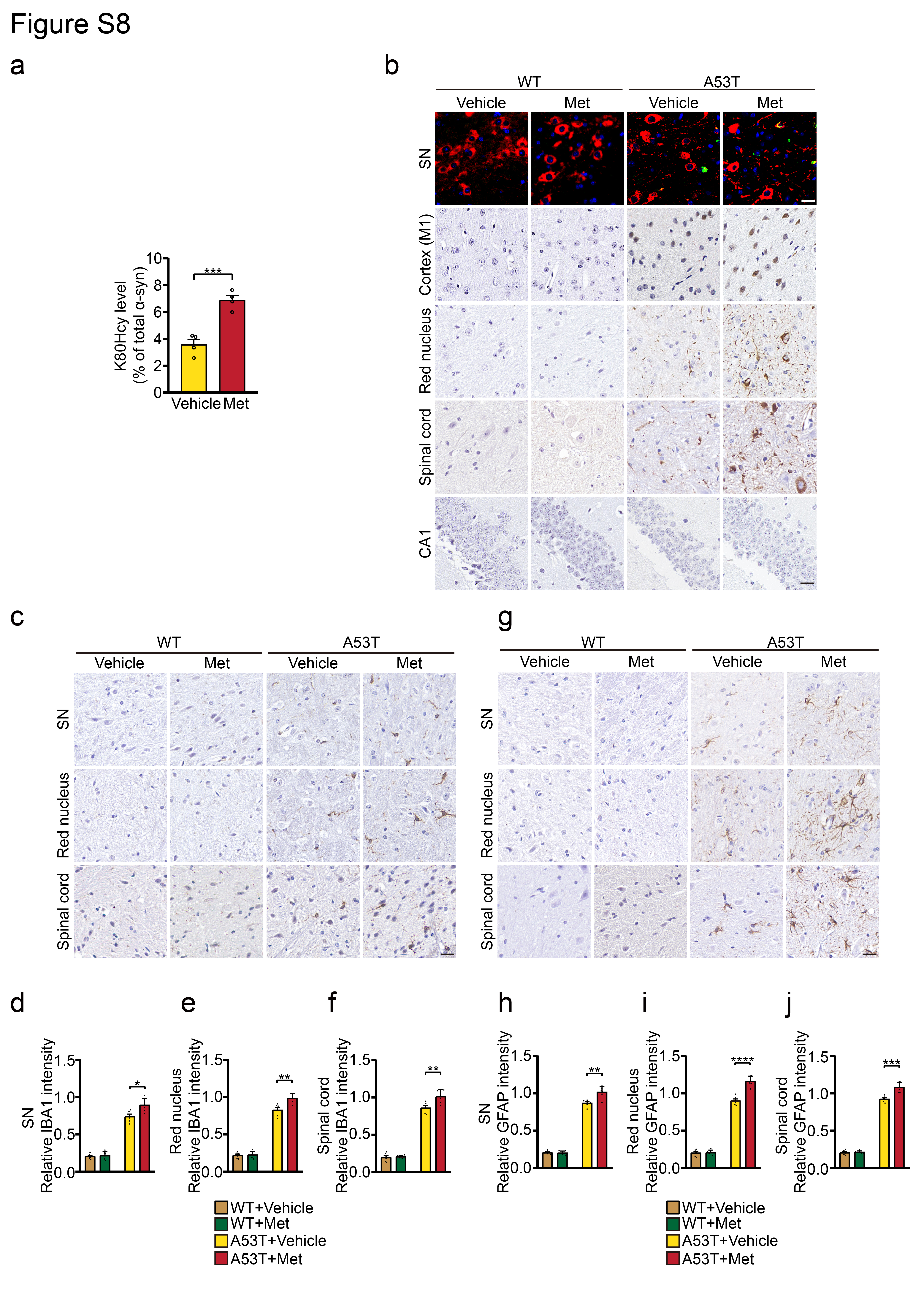
**Figure S8. Met administration enhances pathology in TgA53T mice.**

(**a**) The percentage of α-syn K80Hcy in the brain of vehicle- and Met-treated TgA53T mice. (**b**) Representative pS129 (green) and TH (red) double-immunostaining in the SN, pS129 immunostaining in the cortex (M1), red nucleus, spinal cord, and hippocampus (CA1). Scale bars is 20 μm. (**c**) Representative IBA1 immunostaining in the SN, red nucleus, and spinal cord. Scale bar is 20 μm. (**d-f**) The intensity of IBA1 positive signals. (**g**) Representative GFAP immunostaining in the SN, red nucleus, and spinal cord. Scale bar is 20 μm. (**h-j**) Intensity of GFAP positive signals. Data are shown as mean ± SEM. n = 4 (a), 6 (c-j) mice per group. *P < 0.05, **P < 0.01, ***P < 0.001, ****P < 0.0001.


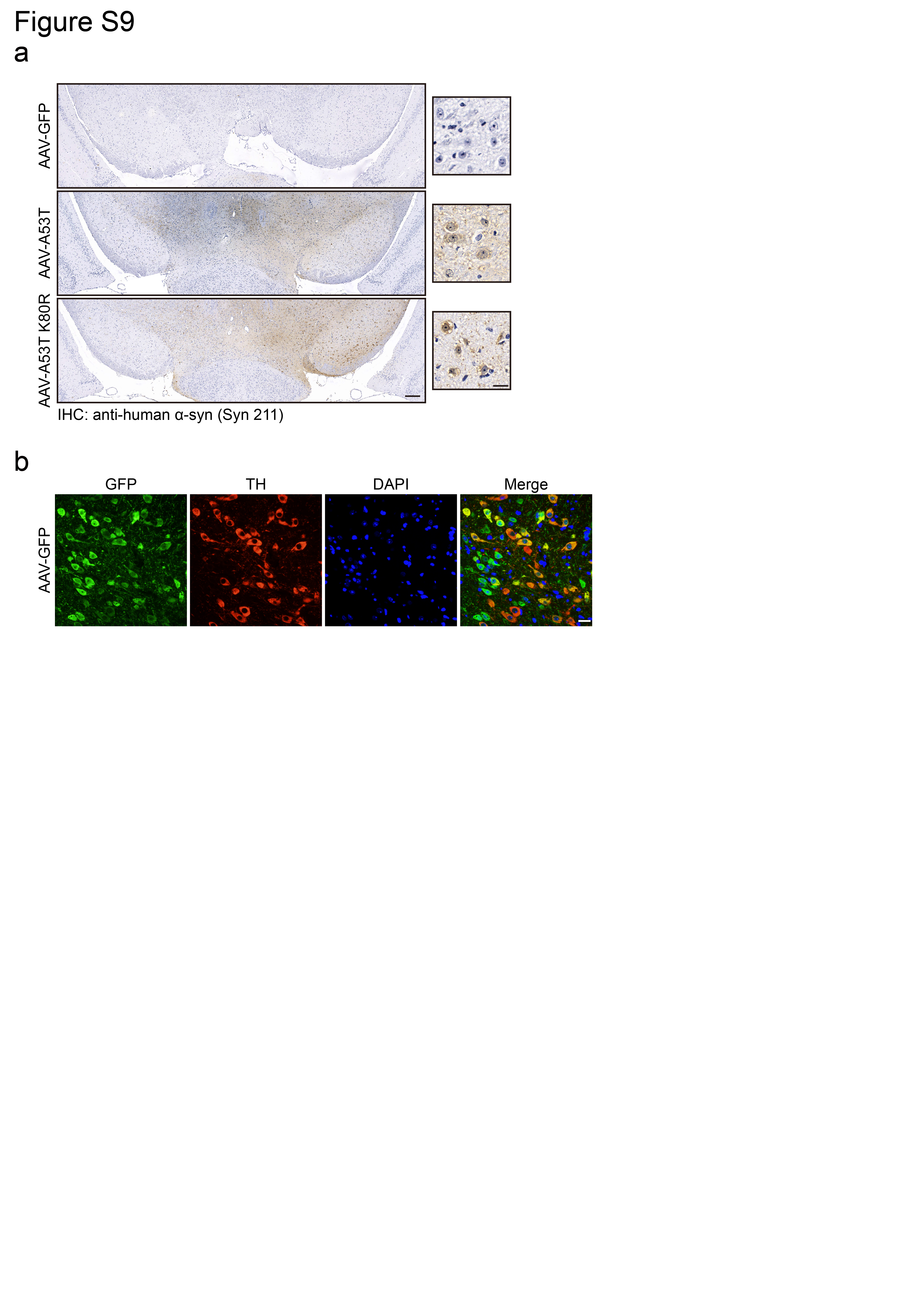


**Figure S9.** **Characterization of α-syn expression in the brain of AAVs-injected mice.**

(**a**) Immunohistochemistry of human α-syn in the SN. Scale bars are 200 μm (left panel) and 20 μm (right panel). (**b**) Representative images showing that AAVs successfully infected the TH-positive neurons of the SNpc. Scale bar is 20 μm.


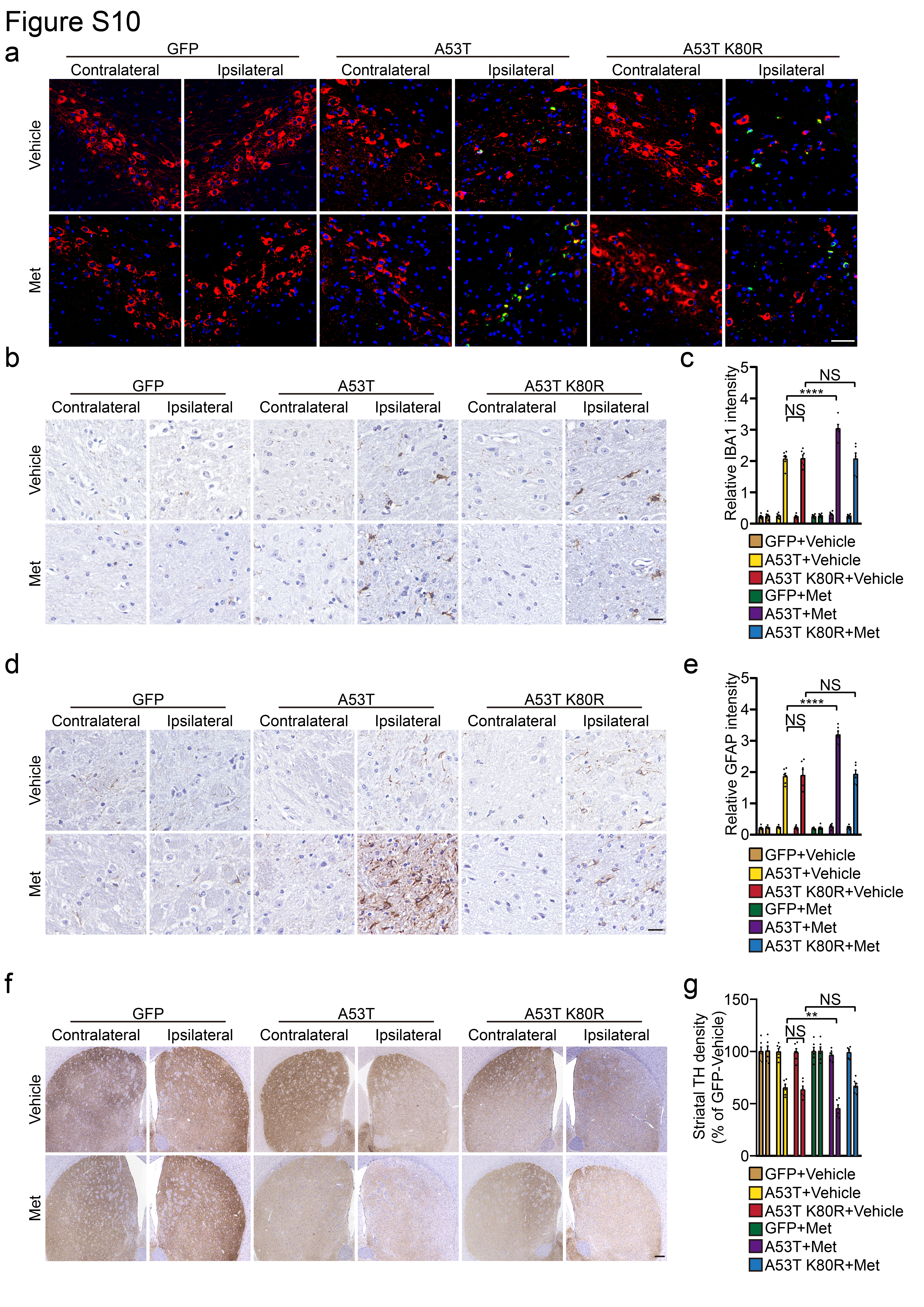
**Figure S10. Blockade of α-syn K80Hcy attenuates α-syn pathology induced by Hcy**

(**a**) Representative double-immunostaining for pS129 (green) and TH (red) in the SN. Scale bar is 50 μm. (**b**) Representative IBA1 immunostaining in the SN. Scale bar is 20 μm. (**c**) Intensity of IBA1 positive signals in the SN. (**d**) Representative GFAP immunostaining in the SN. Scale bar is 20 μm. (**e**) Intensity of GFAP positive signals in the SN. (**f,g**) TH immunohistochemistry in the striatum. Scale bar is 200 μm. Data are shown as mean ± SEM. n = 6 (b-g) mice per group. **P < 0.01, ****P < 0.0001, ^NS^ not significant.

| Peptide position (α-synuclein) | Peptide Sequencea | ptmRS: Best Site Probabilities | XCorr Sequest HTb |
| --- | --- | --- | --- |
| 59-96 | TKEQVTNVGGAVVTGVTAVAQKTVEGAGSIAAATGFVK | K22(Hcy): 100 | 8.29656601 |
| 46-80 | EGVVHGVATVAEKTKEQVTNVGGAVVTGVTAVAQK | K13(Hcy): 50; K15(Hcy): 50 | 5.12337923 |
| 81-102 | TVEGAGSIAAATGFVKKDQLGK | K16(Hcy): 99.76 | 2.729777813 |
| 59-80 | TKEQVTNVGGAVVTGVTAVAQK | K2(Hcy): 100 | 4.878143311 |
| 81-97 | TVEGAGSIAAATGFVKK | K16(Hcy): 99.64 | 4.382165909 |
| 61-97 | EQVTNVGGAVVTGVTAVAQKTVEGAGSIAAATGFVKK | K20(Hcy): 100 | 3.077811956 |
| 61-96 | EQVTNVGGAVVTGVTAVAQKTVEGAGSIAAATGFVK | K20(Hcy): 100 | 2.768387556 |
| 33-43 | TKEGVLYVGSK | K2(Hcy): 100 | 2.797585487 |
| 46-60 | EGVVHGVATVAEKTK | K13(Hcy): 100 | 3.489657402 |
| 13-23 | EGVVAAAEKTK | K9(Hcy): 99.77 | 2.383600473 |
| 44-58 | TKEGVVHGVATVAEK | K2(Hcy): 100 | 4.277832031 |
| 44-60 | TKEGVVHGVATVAEKTK | K15(Hcy): 100 | 2.321553946 |
| 35-45 | EGVLYVGSKTK | K9(Hcy): 100 | 3.089767694 |

**Table S1. Identified homocysteinylated lysine residues on α-syn.**

^a^ Homocysteinylated lysine residues are highlighted.

^b^ Shown are the Sequest XCorr scores for each peptide.

|  | **(Kapp) (h^-1^)** | **Lag time (h)** |
| --- | --- | --- |
| α-Syn | 0.0229 | 92.40 |
| α-Syn+HTL | 0.0545 | 34.93 |
| K80R | 0.0234 | 93.42 |
| K80R+HTL | 0.0236 | 98.48 |

**Table S2. The kinetics parameters of fibrils calculated by ThT fluorescence assay.**
